# The synthetic glucocorticoids prednisolone and dexamethasone regulate the same genes in acute lymphoblastic leukemia cells

**DOI:** 10.1186/1471-2164-15-662

**Published:** 2014-08-07

**Authors:** Daniel Bindreither, Simone Ecker, Barbara Gschirr, Anita Kofler, Reinhard Kofler, Johannes Rainer

**Affiliations:** Division of Molecular Pathophysiology, Biocenter, Medical University Innsbruck, Innrain 80-82, 6020 Innsbruck, Austria; Structural Biology and Biocomputing Program, Spanish National Cancer Research Center (CNIO), Madrid, Spain; Tyrolean Cancer Research Institute, Innrain 66, 6020 Innsbruck, Austria

**Keywords:** Childhood acute lymphoblastic leukemia, Glucocorticoid, Dexamethasone, Prednisolone, Transcriptional response

## Abstract

**Background:**

Glucocorticoids (GCs) cause apoptosis in malignant cells of lymphoid lineage by transcriptionally regulating a plethora of genes. As a result, GCs are included in almost all treatment protocols for lymphoid malignancies, particularly childhood acute lymphoblastic leukemia (chALL). The most commonly used synthetic GCs in the clinical setting are prednisolone and dexamethasone. While the latter has a higher activity and more effectively reduces the tumor load in patients, it is also accompanied by more serious adverse effects than the former. Whether this difference might be explained by regulation of different genes by the two GCs has never been addressed.

**Results:**

Using a recently developed GC bioassay based on a GC-responsive reporter construct in human Jurkat T-ALL cells, we found ~7-fold higher biological activity with dexamethasone than prednisolone. Similarly, 1.0e-7 M dexamethasone and 7.0e-7 M prednisolone triggered similar cell death rates in CCRF-CEM-C7H2 T-chALL cells after 72 hours of treatment. Using microarray-based whole genome expression profiling and a variety of statistical and other approaches, we compared the transcriptional response of chALL cells to 6 hour exposure to both synthetic GCs at the above concentrations. Our experiments did not detect any gene whose regulation by dexamethasone differed significantly from that by prednisolone.

**Conclusions:**

Our findings suggest that the reported differences in treatment efficacy and cytotoxicity of dexamethasone and prednisolone are not caused by inherent differences of the 2 drugs to regulate the expression of certain genes, but rather result either from applying them in biologically in-equivalent concentrations and/or from differences in their pharmacokinetics and - dynamics resulting in different bioactivities in tumor cells and normal tissues.

**Electronic supplementary material:**

The online version of this article (doi:10.1186/1471-2164-15-662) contains supplementary material, which is available to authorized users.

## Background

Acute lymphoblastic leukemia (ALL) is a rapidly-developing aggressive cancer of white blood cells that starts in the bone marrow [1], and is the most prevalent pediatric cancer [2]. Glucocorticoids (GCs) cause massive cell death and cell cycle arrest in malignant cells from the lymphoid lineage and are therefore included in almost all treatment protocols for lymphoid malignancies, particularly childhood acute lymphoblastic leukemia (chALL) [2]. GCs exert their effects through their cognate receptor, the GC-receptor (GR, encoded by the gene *NR3C1*). The GR is a ligand-activated zinc finger transcription factor of the nuclear receptor family [3] that resides in the cytoplasm and, upon ligand binding, translocates into the nucleus, where it modulates expression of its target gene either by binding to GC-responsive elements in the gene promoters/enhancers or by protein-protein interactions with other transcription factors [4].

Treatment of chALL includes administration of synthetic GCs, mostly prednisolone (PRED), but also dexamethasone (DEX), during various phases of therapy in combination with chemotherapeutic drugs, resulting in a cure rate of up to ~90% [2, 5]. The most commonly used GC in chALL is PRED, the active metabolite of prednisone [6]. In the past several years, DEX has been increasingly used for chALL treatment, specifically in a delayed intensification phase of current treatment protocols [7] that result in lower bone marrow and CNS relapse rates, but also increased adverse effects. DEX differs molecularly from PRED only by a fluorine atom at the 9α position of ring B and a methyl group at position C16 of ring D. DEX has enhanced lymphoblast cytotoxicity and CNS penetration capability, the latter being crucial to successful ALL therapy since, despite the good outcome of contemporary childhood ALL treatments, CNS relapses remain a challenge [6]. Treatment of the CNS is considered a quantum leap forward in improving the overall survival of chALL patients [8]. Pharmacokinetic investigations showed that the shorter half-life of PRED (60 minutes for PRED *vs.* 200 minutes for DEX, biological half-lives 24–36 *vs.* 36–54 hours [9]) affects its protein-binding properties and might reduce the duration of leukemic cell exposure to cytotoxic concentrations in the cerebrospinal fluid, compromising its effectiveness [6, 10]. Moreover, the DEX-GR complex is thought to be more stable than the PRED-GR complex, and the GR seems to have a greater affinity for DEX than PRED in leukemic cells [11]. Generally, DEX seems to be the more active corticosteroid in the treatment of ALL [12, 13], and many studies reported increased event-free survival and significantly decreased risk of CNS relapses with DEX vs. PRED [5–7].

However, these improvements are also associated with increased toxicity of DEX. Specifically, a symptomatic osteonecrosis that disproportionately affects adolescent chALL patients has been correlated with continuous DEX treatment in the delayed intensification phase [14]. Lowering the effective DEX concentration by alternative week rather than continuous treatment in a 21 day-long delayed intensification phase has been shown to significantly reduce osteonecrosis among high risk chALL patients (specifically those over 16 years of age) [14].

Analysis of DEX and PRED data from the literature is complicated due to heterogeneity of treatment regimens, dose ratios and study populations. Teuffel et al. [15] recently published a meta-analysis comparing the efficacy and toxicity of both synthetic GCs in the induction phase of chALL therapy in an effort to account for the above mentioned complications. While DEX has been shown to be more effective than PRED in lowering CNS and bone marrow relapses, it was also significantly associated with death during induction, neuro-psychiatric adverse events and myopathy.

Although many clinical studies comparing the effects of PRED and DEX have been conducted, to our knowledge, the differences between the two GCs have not been investigated on a molecular level. This study, therefore, focused on determining whether these differences might be explained by differences in the transcriptional responses of chALL cells to treatment with the 2 synthetic GCs at comparable biological activities.

## Results

### Bioactivity of dexamethasone and prednisolone

**Figure 1:**
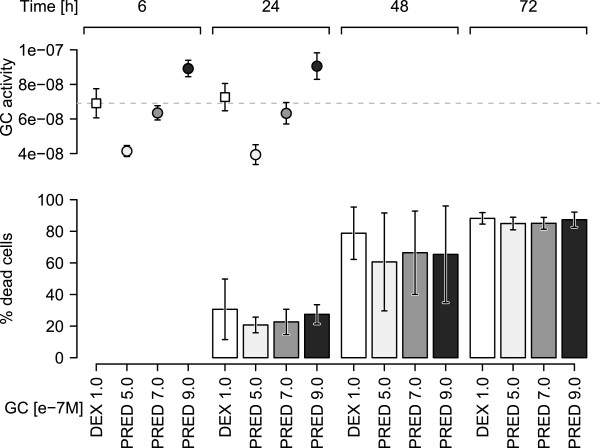
**GC bioactivity and GC-induced apoptosis.** Upper panel: GC-bioactivity in cell supernatants of dexamethasone (rectangles) and prednisolone (circles) for the indicated time points and concentrations. Shown are mean and standard deviations from 3 independent experiments. Lower panel: Percentages (mean and standard deviation from 3 independent experiments) of dead cells after exposure to DEX or PRED at the indicated time points.

Comparison of the transcriptional responses to the two synthetic GCs requires treatment of the cells in bio-equivalent concentrations of the two agents. The commonly used concentration of DEX in *in vitro* studies is 1.0e-7 M [16, 17]. To determine an equivalent PRED concentration, we treated T-chALL CCRF-CEM-C7H2 cells in three independent experiments with 1.0e-7 M DEX and 5.0e-7 M, 7.0e-7 M and 9.0e-7 M PRED concentrations. Ethanol-treated cells served as empty carrier controls. GC-bioactivity levels were recorded in all experiments after 6 and 24 hours of treatment in the cell supernatants using our recently established GC-bioactivity assay (GBA) [18]. The GBA measures GC activity using a reporter construct containing GR binding sites, thus directly determining the transcriptional activity of the ligand-activated GR. In addition, cells were harvested after 24, 48 and 72 hours, treated with propidium iodide (PI) and subjected to FACS analysis to determine the percentage of cells undergoing cell death. GC-bioactivity levels remained constant over time for all GCs/concentrations, suggesting a long half-life of PRED and DEX (Figure 1, upper panel). From all PRED concentrations used, 7.0e-7 M yielded the closest GC-bioactivity to 1.0e-7 M (see Figure 1, upper panel) and was thus selected as the DEX-equivalent PRED concentration. Cell death induction was observed for all GC concentrations, with rates surpassing 80% after 72 hours in all instances. Interestingly, however, cell death rates of all PRED concentrations were slightly lower after 48 hours than those of 1.0e-7 M DEX.

To determine whether the GC activity of these concentrations is achievable in children given the standard scheduling and dosing of the drugs we measured the GC bioactivity in serum samples of 8 childhood B-ALL patients taken prior to and 6 and 24 hours after initiation of the systemic GC mono-therapy. The administered prednisolone resulted in GC bioactivities equivalent to that of 1.0e-7 M DEX (see Additional file 1: Figure S2), showing that the *in vitro* used concentrations are indeed pharmacologically relevant.Taken together, 7.0e-7 M PRED resulted in about the same GC bioactivity as 1.0e-7 M DEX and had a similar potency to induce cell death in ALL cells (Figure 1, lower panel).

### Comparison of the transcriptional responses to DEX and PRED

We next investigated whether the response of ALL cells exposed to either synthetic GC differed at the transcriptional level by generating a microarray data set with RNA extracted from cells of the above experiments, i.e., from CCRF-CEM-C7H2 cells treated for 6 hours with 1.0e-7 M dexamethasone, 7.0e-7 M prednisolone (the DEX-equivalent prednisolone concentration determined above) or 0.1% ethanol as empty carrier control. Thus the data set consisted of a total of 9 Affymetrix Gene ST 1.0 GeneChips with 3 biological replicates for each condition. Even though cell death occurs considerably later (see Figure 1) we selected the 6 hours time point, because we were particularly interested in the early initiation of transcriptional response ultimately leading to cell death. Also, most of the GC-regulated genes after 6 hours are potential direct GR target genes, as suggested by the presence of GR binding sites in their promoter/enhancer regions [17].

**Figure 2:**
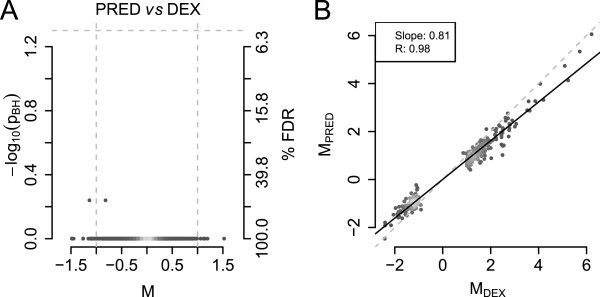
**Direct comparison of the transcriptional responses to DEX and PRED exposure. A**: Volcano plot showing the difference in GC regulation (M, i.e. log2 fold-change) for each gene against its significance (-log10 of the p-values adjusted for multiple hypothesis testing) between 6 hours prednisolone (PRED) or dexamethasone (DEX) treatments. The grey-dashed horizontal line represents the significance cut-off (5% FDR), the dashed vertical lines the cut-off for the extent of regulation (two-fold difference). Points are colored according to the local point density. **B**: Correlation of M-values representing the extent of gene regulation by DEX or PRED treatment. Shown are genes identified to be significantly regulated by either DEX or PRED treatment. The grey-dashed and the solid black line represent the identity line and the linear regression fit to the data, respectively.

First, we directly compared the PRED- and DEX-treated samples. Gene-wise tests for differential regulation were conducted as paired tests based on the observation of different apoptosis rates between the experiments (see Additional file 1: Figure S3). Criteria for significant differences in all comparisons were an adjusted p-value < 0.05 and an absolute M-value (log2 fold-change) > 1. Genes passing these criteria were more than two-fold regulated at a 5% false discovery rate (FDR). In this analysis, not a single gene was found significantly differently regulated between PRED and DEX treated samples (Figure 2A). Thus, using this approach, there was no significant difference in the transcriptional response of C7H2 ALL cells after exposure to either of the 2 synthetic GCs.

In an alternative approach, we correlated the average M-values for DEX and PRED treatment, determined by comparisons of GC- to ethanol-exposed cells and representing GC-regulation of all genes by either synthetic GC. A high correlation of GC-regulations was observed (R = 0.84, Additional file 1: Figure S4) that was even higher when only genes found to be significantly regulated by one of the two GCs using the above cut-off criteria (R = 0.98, Figure 2B) were considered. The slope of the linear regression line fit to the PRED *versus* DEX data was 0.81, suggesting that the overall response was slightly stronger (~1.2 fold) in DEX- compared to PRED-treated samples (see Figure 2B). This observation is in good agreement with the slightly lower GC-bioactivity measured for 7.0e-7 M PRED (see Figure 1), and also with the slightly lower cell death rates after 48 hours (Figure 1B). Thus, this alternative approach further supported the notion that both drugs regulate the same genes.

**Figure 3:**
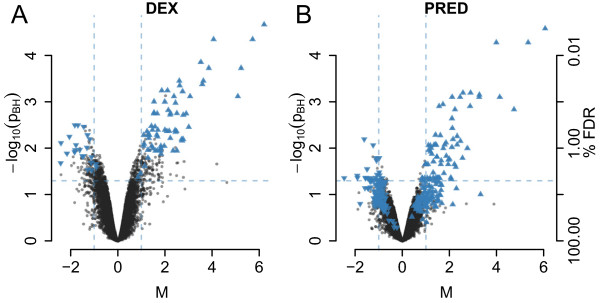
**Comparison of DEX and PRED-regulated genes.** Volcano plots showing the extent and significance of GC regulation after 6 hours treatment with DEX **(A)** or PRED **(B)**. Genes significantly regulated by PRED are highlighted by blue triangles in the volcano plot for DEX treatment and *vice versa*, with triangles pointing up- or downwards indicating up- or down-regulations, respectively. The dashed horizontal and vertical lines represent the cut-off criteria for significant regulation.

**Figure 4:**
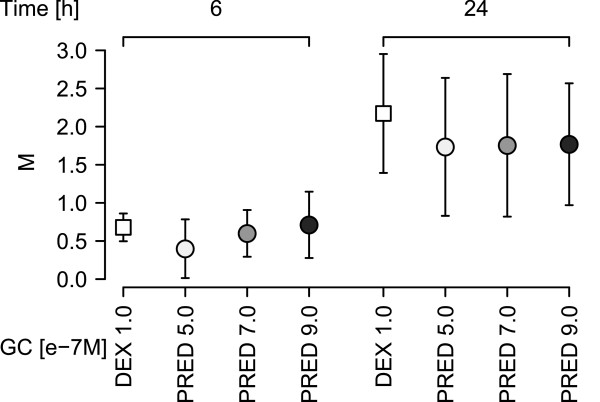
**GC-regulation of IL6ST.** Shown are mean M-values (log2-fold change values) and standard deviations for 6 or 24 hours DEX and PRED-treated CCRF-CEM-C7H2 cells in the indicated concentrations, representing GC-regulation of IL6ST measured by real time RT-PCR in triplicates in 3 independent experiments.

Finally, we applied an approach that is frequently used but, as mentioned below and detailed in the Supplement, has significant inherent problems. This analysis compares genes found to be significantly regulated by either treatment. Again, we used paired statistics to test for significance of differential expression between GC-treated and control samples and applied the above cut-off criteria to define genes as being either DEX- or PRED-regulated. In this comparison, we found what first appeared to be quite remarkable differences, i.e., 93 PRED-regulated *versus* 295 DEX-regulated genes (see Additional file 1: Figure S6). However, as detailed in the supplement, this difference probably resulted from technical and/or analytical issues. Thus, the M- and/or p-values for 181 of the 208 genes found to be significantly regulated by only one of the two GCs were just below the cut-off for the other GC (see also Figure 3). A heatmap representing the results of a hierarchical cluster analysis on the per-experiment M-values of the above genes also showed that DEX-regulated genes are concordantly regulated by PRED and *vice versa*, albeit to a slightly lower extent (see Additional file 1: Figure S7). Therefore, such genes cannot be considered “unregulated” by the respective GC. These “borderline regulations” might be explained (at least in part) by the slightly lower bioactivity of the PRED concentration used (Figure 1 upper panel and Figure 2B) and the higher inter-replicate variance in the PRED-treated cells (see Additional file 1: Figure S5), resulting in lower significance levels. The remaining 21 genes corresponded quite well to the expected number of false positives (5% FDR for 295 genes = ~15). To test this possibility, we analyzed a completely independent set of microarrays with RNA from C7H2 cells treated for 6 hours in 3 independent experiments with either 1.0e-7 M DEX, 4.0 or 8.0e-6 M Solu-dacortin (a water soluble form of prednisolone) or 0.1% ethanol. In this data set, 17 of the 21 genes were not significantly regulated by DEX (see Additional file 1: Table S4). Two of the remaining 4 genes were also regulated by Solu-dacortin. Real-time RT-PCR analysis of one of the remaining 2 genes (IL6ST) revealed that this gene was induced both by DEX and PRED (see Figure 4). The increasing up-regulation of this gene with increasing GC concentrations along with the increasing bioactivity measured by the GBA for higher PRED concentrations (Figure 1) suggested a dosage dependency of the transcriptional response to GCs. Summarizing, this elaborate analysis provided no evidence that DEX and PRED regulated different genes, and also clearly highlighted the problems of this type of microarray data analysis.

We next evaluated the GC-regulation of the GR (NR3C1) and of known GR target genes TSC22D3 (also known as GILZ), FKBP5, RCAN1, SOCS1 and DDIT4 [17, 19–21]. All of these genes were significantly and strongly up-regulated by DEX and by PRED (see Additional file 1: Table S5). The extent of up-regulation was highly similar which further suggests that the employed concentrations result in equivalent transcriptional activities.

In conclusion, the combined data strongly suggest that DEX and PRED regulated the same genes, at least in the investigated chALL cell line model.

## Discussion

This report addressed the clinically and biologically relevant question of whether the observed differences in clinical outcomes in chALL patients treated with either DEX or PRED might be explained by a difference in the genes regulated by either synthetic GC. Such a difference could result from the fact that different ligands might generate distinct platforms on the GR for interaction with co-regulatory proteins, as has been suggested for numerous “discriminatory” GR ligands that may mediate gene regulation *via* transactivation rather than transrepression or *vice versa* (for review see [22]). If so, the differentially regulated genes could be easily identified in an approach such as ours, which might then lead to novel therapeutic measures directed at manipulating the respective pathways. However, our comprehensive analysis did not identify any significant differences in the transcriptional response of ALL cells to treatment with either synthetic GC. Thus, our findings strongly suggest that the differential effects of the 2 synthetic GCs, at least with regard to their efficacy, do not result from differences in gene regulations. The increased efficacy of DEX over PRED may rather result from applying PRED at lower biological activity, better transfer of DEX through the blood–brain barrier [6], a longer half-life time of DEX [9] or other differences in pharmacokinetics and/or pharmacodynamics. Concerning adverse effects, our study cannot provide a corresponding conclusive answer because these effects primarily result from the effect of GCs on other tissues in the body that have not been investigated. Given that the same genes are regulated in the chALL cell system analyzed here, it seems unlikely that the transcriptional response to treatment with the 2 GCs differs in other tissues although this possibility has not been formally ruled out.

Interestingly, from a more technical point of view, while DEX- and PRED-treated cells showed no significant differences in gene regulations (Figure 2A), and correlation of M-values failed to reveal differences in the transcriptional response of ALL cells to either synthetic GC (Figure 2B), the responses seemed to differ when comparing genes defined as significantly GC-regulated by each individual GC (Additional file 1: Figure S6). However, this apparent difference was misleading: as we demonstrated, such an approach to compare transcriptional responses, or differences between microarray results in general, which is based simply on the significantly-regulated genes, is highly problematic because of its strong dependency on the defined hard cut-off criteria for gene regulation and on properties of the data set itself, such as inter-replicate variances. In our setting, the slightly weaker bioactivity of the PRED concentration used and the higher inter-replicate variance in the PRED samples resulted in a lower number of significantly regulated genes compared to DEX treatment. More thorough analysis of the genes found to be significantly regulated only by DEX revealed that the differences were either quantitative or corresponded to false positives. Thus, comparison of actual gene regulation values by both treatments using statistical approaches and correlation of these M-values are clearly superior in such a setting since they directly compare gene regulations caused by treatment with either synthetic GC.

## Conclusions

In this study, we compared the transcriptional response of childhood T-ALL cells to treatment with the synthetic GCs prednisolone and dexamethasone. Based on data from a total of 21 microarrays and real-time RT-PCR-based verifications, we observed no significant differences in responses, and concluded that both GCs regulate the same genes in chALL cells. The slightly weaker response of PRED-treated cells was probably caused by the slightly lower activity of 7.0e-7 M PRED compared to 1.0e-7 M DEX.

Our findings suggest that the reported differences in treatment efficacy and cytotoxicity of dexamethasone and prednisolone are not caused by inherent differences of the 2 drugs to regulate the expression of certain genes, but rather result either from applying them in biologically in-equivalent concentrations and/or from differences in their pharmacokinetics and -dynamics resulting in different bioactivities in tumor cells and normal tissues.

## Methods

### Cell system and GC-treatment

We used the GC-sensitive C7H2 sub-clone of the extensively studied childhood T-ALL cell line CCRF-CEM [23–26] to evaluate GC-activity, apoptosis induction and gene regulations induced by dexamethasone or prednisolone treatment. Cells were kept in culture in RPMI 1640 supplemented with 10% fetal calf serum and 2 mM L-glutamine at 37°C, 5% CO_2_ and saturated humidity. For gene expression profiling, apoptosis determination and GC-bioactivity experiments, the cells were cultured in the presence of 1.0e-7 M dexamethasone and varying concentrations of prednisolone, i.e. 5.0, 7.0 and 9.0e-7 M (with 7.0e-7 M of PRED being used for gene expression profiling). Both GCs were solved in ethanol, thus, cells treated with 0.1% of ethanol were used as empty carrier controls. In additional experiments, C7H2 cells were treated with 1.0e-7 M dexamethasone, 2.0, 4.0, 6.0 and 8.0 e-6 M Solu-dacortin [prednisolone 21-(sodium succinate), a water-soluble prednisolone], and 2.0, 4.0 and 6.0 e-7 M Dexabene [dexamethasone 21-(disodium phosphate); a water-soluble dexamethasone]. GC-bioactivity was measured after 6, 24 and 48 hours of treatment, and cell death induction after 24, 48 and 72 hours. To determine in vivo GC bioactivity of PRED during systemic GC mono-therapy we performed GBA measurements on serum samples of previously published GC-treated patients with childhood ALL [16].

### Cell death determinations

Apoptosis was determined by fluorescence-activated cell sorter (FACS) analyses of propidium iodide (PI)-treated permeabilized cells, as previously detailed [27]. In brief, cells were analyzed with a FACScan cytometer (Becton Dickinson Biosciences, San Jose, CA) to acquire forward scatter/sideward scatter and FL-2 (log scale). In FL-2, the percentage of nuclei with reduced DNA-content (sub-G1 peak) was assessed and reported as percentage of dead cells.

### GC bioactivity measurements

Activity of the administered GCs was estimated using our recently-developed GC bioactivity assay (GBA) [18], which measures transcriptional activity of the GR upon GC-treatment using a reporter construct containing a known GR binding site. Aliquots of 9.0e4 cells containing the reporter construct in 100 μL culture medium were supplemented either with a log2 step DEX dilution series in 25 μL of culture medium as standard or with 25 μL of supernatant from the DEX- and PRED-treated cells in their respective concentrations. These samples were plated in 96-well plates and incubated at 37°C and 5% CO_2_ in a humidified incubator. After an incubation of 6 hours, mean Venus nuclear protein (VNP) activity was determined analyzing 20,000 events and measuring mean fluorescence in the FL-2 channel with a FACScan cytometer. Cell debris was removed from the analysis. Mean VNP activity values from samples with unknown GC bioactivity were fit to the linear part of the VNP values from the standard curve by dilution and the corresponding GC-activity (expressed in DEX equivalents) was reported.

### Microarray data set generation and pre-processing

Microarray data generation was performed according to the manufacturer’s protocols. In brief, total cell RNA was extracted using the TRIZOL protocol, and RNA quantity and integrity was determined by optical density measurements and the 2100 Bioanalyzer (Agilent Technologies, Palo Alto, CA), respectively. Two hundred and fifty ng of high quality RNA was processed using the Ambion Affymetrix GeneChip WT Expression Kit (Part no. 4411974, Ambion) and the Affymetrix GeneChip WT Terminal Labeling Kit (Affymetrix). The resulting biotinylated targets were hybridized in an Affymetrix hybridization oven to a total of 9 Affymetrix Human Gene ST 1.0 GeneChips. The microarrays were washed and stained in an Affmetrix fluidic station 450 and fluorescence signals were recorded in an Affymetrix scanner 3000. All further analysis was performed in R (version 3.0.2) using packages from the Bioconductor project ([28], version 2.13). The raw microarray data was pre-processed using the “generalgcrma” package [17] and our custom transcript-level “CEL definition file” (CDF) that defines probe sets for each transcript of all genes defined in the Ensembl database version 74. Generation of the CDF package for the analysis is similar to that reported by Rainer and colleagues [17] except that the 25 nt long probe sequences were aligned against the cDNA sequences and sequences of non-coding transcripts. The CDF defined a total of 125,709 transcript probe sets for 25,426 genes. After GCRMA [29] pre-processing, a representative transcript probe set was selected for each gene. Similar to the study of Aneichyk et al. [30], transcript probe sets were preferred consisting of more than 9 probes and with high average expression and variance in expression across all 9 samples.

Generation and pre-processing of the second microarray data set of 12 Affymetrix Human Gene ST microarrays with RNA from C7H2 cells treated with 6 hours 1.0e-7 M DEX, 4.0 and 8.0e-6 M Solu-dacortin, and 6 hours of ethanol treatment was performed analogously except that for cRNA cleanup of the 3 GeneChips for 8.0e-6 M Solu-dacortin treatment, the Affymetrix GeneChip 3′ IVT Expression Kit was used.

Raw and pre-processed microarray data has been deposited at the Gene Expression Omnibus (Accession number GSE55878).

### Differential expression analysis

For differential expression analysis, we used the moderated t-test implemented in Bioconductor’s limma package [31] and subsequently adjusted the obtained p-values using the multiple hypotheses testing correction method of Benjamini and Hochberg (BH) [32] for strong control of the false discovery rate (FDR). Based on the observation that apoptosis rates differed between experiments after 24 and 48 hours (see Additional file 1: Figure S3), we included a categorical variable representing the experiment assignment of the sample into the linear regression model used for the differential expression analysis, rendering the resulting tests equivalent to paired tests. To assess differences in transcriptional responses, we directly compared PRED- and DEX-treated samples. To identify genes significantly regulated by each synthetic GC, we compared DEX- and PRED-treated samples to the empty carrier controls (ethanol-treated samples).

We considered genes with an adjusted p-value smaller than 0.05 and an absolute M-value (log2 fold-change) or difference in M-values greater than 1 as significantly differentially expressed or regulated. This cut-off controls the false discovery rate (FDR) at 5% and additionally requires at least a 2-fold regulation (or difference in regulation) of a gene.

### Real-time RT-PCR

Samples were taken at the time points indicated in the text and subjected to AB Taqman-based real-time RT-PCR similar to that reported by Mansha et al. [33] using the following assays: TBP: Hs00427620_m1, IL6ST: Hs01006739_m1 (detecting all RefSeq and most GenBank mRNAs of IL6ST).

Measurements were performed in the same RNA samples used for microarray-based gene expression profiling. CT values of three technical replicates were averaged and normalized against the housekeeping gene TATA-box binding protein (TBP). Normalized measurements (ΔCT) of the 3 biological replicates were averaged and the difference between GC-treated and control samples were reported as M-values (log2-fold change values, -ΔΔCT).

## Availability of supporting data

The data sets supporting the results of this article are included within the article (and its additional files). Raw and pre-processed microarray data is available at the Gene Expression Omnibus with accession numbers GSE55877 (main data set) and GSE55876 (additional data set with cells exposed to Solu-dacortin, a water soluble form of PRED) and GSE55878 for the full series.

## Electronic supplementary material

Additional file 1: **Supplementary Figures and Tables.** (PDF 1 MB)

## Abbreviations

chALL: Childhood acute lymphoblastic leukemia
CNS: Central nervous system
DEX: Dexamethasone
GBA: Glucocorticoid bioactivity assay
GC: Glucocorticoid
FDR: False discovery rate
PI: Propidium iodide
PRED: Prednisolone
VNP: Venus nuclear protein.

## References

[1] Stanulla M, Schrappe M (2009). Treatment of childhood acute lymphoblastic leukemia. Semin Hematol.

[2] Pui C-H, Robison LL, Look AT (2008). Acute lymphoblastic leukaemia. Lancet.

[3] Laudet V, Gronemeyer H (2002). The Nuclear Receptor Factsbook.

[4] Kassel O, Herrlich P (2007). Crosstalk between the glucocorticoid receptor and other transcription factors: molecular aspects. Mol Cell Endocrinol.

[5] Inaba H, Greaves M, Mullighan CG (2013). Acute lymphoblastic leukaemia. Lancet.

[6] Inaba H, Pui C-H (2010). Glucocorticoid use in acute lymphoblastic leukaemia. Lancet Oncol.

[7] McNeer JL, Nachman JB (2010). The optimal use of steroids in paediatric acute lymphoblastic leukaemia: no easy answers. Br J Haematol.

[8] Norris RE, Adamson PC (2012). Challenges and opportunities in childhood cancer drug development. Nat Rev Cancer.

[9] Meikle AW, Tyler FH (1977). Potency and duration of action of glucocorticoids. Effects of hydrocortisone, prednisone and dexamethasone on human pituitary-adrenal function. Am J Med.

[10] Balis FM, Lester CM, Chrousos GP, Heideman RL, Poplack DG (1987). Differences in cerebrospinal fluid penetration of corticosteroids: possible relationship to the prevention of meningeal leukemia. J Clin Oncol.

[11] Iacobelli S, Natoli V, Longo P, Ranelletti FO, De Rossi G, Pasqualetti D, Mandelli F, Mastrangelo R (1981). Glucocorticoid receptor determinations in leukemia patients using cytosol and whole-cell assays. Cancer Res.

[12] Kaspers GJ, Kaspers GJL, Veerman AJP, Veerman AJ, Popp-Snijders C, Lomecky M, Van Zantwijk CH, Swinkels LM, Swinkels LMJW, Van Wering ER, Pieters R (1996). Comparison of the antileukemic activity in vitro of dexamethasone and prednisolone in childhood acute lymphoblastic leukemia. Med Pediatr Oncol.

[13] Cantrill HL, Waltman SR, Palmberg PF, Zink HA, Becker B (1975). In vitro determination of relative corticosteroid potency. J Clin Endocrinol Metab.

[14] Mattano LA, Devidas M, Nachman JB, Sather HN, Hunger SP, Steinherz PG, Gaynon PS, Seibel NL, Children’s Oncology Group (2012). Effect of alternate-week versus continuous dexamethasone scheduling on the risk of osteonecrosis in paediatric patients with acute lymphoblastic leukaemia: results from the CCG-1961 randomised cohort trial. Lancet Oncol.

[15] Teuffel O, Kuster SP, Hunger SP, Conter V, Hitzler J, Ethier M-C, Shah PS, Beyene J, Sung L (2011). Dexamethasone versus prednisone for induction therapy in childhood acute lymphoblastic leukemia: a systematic review and meta-analysis. Leukemia.

[16] Schmidt S, Rainer J, Riml S, Ploner C, Jesacher S, Achmüller C, Presul E, Skvortsov S, Crazzolara R, Fiegl M, Raivio T, Jänne OA, Geley S, Meister B, Kofler R (2006). Identification of glucocorticoid-response genes in children with acute lymphoblastic leukemia. Blood.

[17] Rainer J, Lelong J, Bindreither D, Mantinger C, Ploner C, Geley S, Kofler R (2012). Research resource: transcriptional response to glucocorticoids in childhood acute lymphoblastic leukemia. Mol Endocrinol.

[18] Oppl B, Kofler A, Schwarz S, Rainer J, Kofler R (2011). Establishing a sensitive and specific assay for determination of glucocorticoid bioactivity. Wien Klin Wochenschr.

[19] Cannarile L, Zollo O, D’Adamio F, Ayroldi E, Marchetti C, Tabilio A, Bruscoli S, Riccardi C (2001). Cloning, chromosomal assignment and tissue distribution of human GILZ, a glucocorticoid hormone-induced gene. Cell Death Differ.

[20] Shen L, Oshida T, Miyauchi J, Yamada M, Miyashita T, U M (2004). Identification of novel direct transcriptional targets of glucocorticoid receptor. Leukemia.

[21] Hirakawa Y, Nary L, Medh R (2009). Glucocorticoid evoked upregulation of RCAN1-1 in human leukemic CEM cells susceptible to apoptosis. J Mol Signal.

[22] Kumar R, McEwan IJ (2012). Allosteric modulators of steroid hormone receptors: structural dynamics and gene regulation. Endocr Rev.

[23] Strasser-Wozak EM, Hattmannstorfer R, Hála M, Hartmann BL, Fiegl M, Geley S, Kofler R (1995). Splice site mutation in the glucocorticoid receptor gene causes resistance to glucocorticoid-induced apoptosis in a human acute leukemic cell line. Cancer Res.

[24] Thompson EB, Johnson BH (2003). Regulation of a distinctive set of genes in glucocorticoid-evoked apoptosis in CEM human lymphoid cells. Recent Prog Horm Res.

[25] Lambrou GI, Vlahopoulos S, Papathanasiou C, Papanikolaou M, Karpusas M, Zoumakis E, Tzortzatou-Stathopoulou F (2009). Prednisolone exerts late mitogenic and biphasic effects on resistant acute lymphoblastic leukemia cells: Relation to early gene expression. Leuk Res.

[26] Laane E, Panaretakis T, Pokrovskaja K, Buentke E, Corcoran M, Söderhäll S, Heyman M, Mazur J, Zhivotovsky B, Porwit A, Grandér D (2007). Dexamethasone-induced apoptosis in acute lymphoblastic leukemia involves differential regulation of Bcl-2 family members. Haematologica.

[27] Geley S, Hartmann BL, Hattmannstorfer R, Löffler M, Ausserlechner MJ, Bernhard D, Sgonc R, Strasser-Wozak EM, Ebner M, Auer B, Kofler R (1997). p53-induced apoptosis in the human T-ALL cell line CCRF-CEM. Oncogene.

[28] Gentleman RC, Carey VJ, Bates DM, Bolstad B, Dettling M, Dudoit S, Ellis B, Gautier L, Ge Y, Gentry J, Hornik K, Hothorn T, Huber W, Iacus S, Irizarry R, Leisch F, Li C, Maechler M, Rossini AJ, Sawitzki G, Smith C, Smyth G, Tierney L, Yang JYH, Zhang J (2004). Bioconductor: open software development for computational biology and bioinformatics. Genome Biol.

[29] Wu Z, Irizarry RA, Gentleman R, Martinez-Murillo F, Spencer F (2004). A model-based background adjustment for oligonucleotide expression arrays. J Am Stat Assoc.

[30] Aneichyk T, Bindreither D, Mantinger C, Grazio D, Goetsch K, Kofler R, Rainer J (2013). Translational profiling in childhood acute lymphoblastic leukemia: no evidence for glucocorticoid regulation of mRNA translation. BMC Genomics.

[31] Smyth GK (2004). Linear models and empirical Bayes methods for assessing differential expression in microarray experiments. Stat Appl Genet Mol Biol.

[32] Benjamini Y, Hochberg Y (1995). Controlling the false discovery rate: a practical and powerful approach to multiple testing. J Roy Statist Soc Ser B.

[33] Mansha M, Carlet M, Ploner C, Gruber G, Wasim M, Wiegers GJ, Rainer J, Geley S, Kofler R (2010). Functional analyses of Src-like adaptor (SLA), a glucocorticoid-regulated gene in acute lymphoblastic leukemia. Leuk Res.

