# Supplementary material for: The synthetic glucocorticoids prednisolone and dexamethasone regulate the same genes in acute lymphoblastic leukemia cells
Source: BMC Genomics. 2014 Aug 7;15(1):662. doi: 10.1186/1471-2164-15-662 (PMC4133055; doi:10.1186/1471-2164-15-662)
Supplement: Supplementary file 1 — Additional file 1: Supplementary Figures and Tables. (PDF 1 MB) [file 12864_2014_6349_MOESM1_ESM.pdf]

Supplementary Information for

The synthetic glucocorticoids prednisolone and dexamethasone regulate the same genes in acute lymphoblastic leukemia cells

Daniel Bindreither[1,4], Simone Ecker[2,3,4], Barbara Gschirr[1,3], Anita Kofler[1,3], Reinhard Kofler[1,3] and Johannes Rainer[1,3,*]

[1] Division of Molecular Pathophysiology, Biocenter, Medical University Innsbruck, Innrain 80-82, 6020 Innsbruc, Austria.
[2] Structural Biology and Biocomputing Program, Spanish National Cancer Research Center (CNIO), Madrid, Spain.
[3] Tyrolean Cancer Research Institute, Innrain 66, 6020 Innsbruck, Austria.
[4] Contributed equally to this work.
[*] Corresponding author: Johannes Rainer, johannes.rainer@i-med.ac.at

Contents

GC-bioactivity and apoptosis induction **1**

Supplementary Figure S1. 1
Supplementary Figure S2. 2
Supplementary Figure S3. 2

Comparison of transcriptional responses **3**

Correlation of M-values 3
Supplementary Figure S4. 3
Comparison of GC-regulated genes 3
Supplementary Figure S5. 3
Supplementary Figure S6. 4
Supplementary Figure S7. 5
Supplementary Table S1. 7
Supplementary Table S2. 11
Supplementary Table S3. 11
Supplementary Table S4. 12
Supplementary Table S5. 12

GC-bioactivity and apoptosis induction

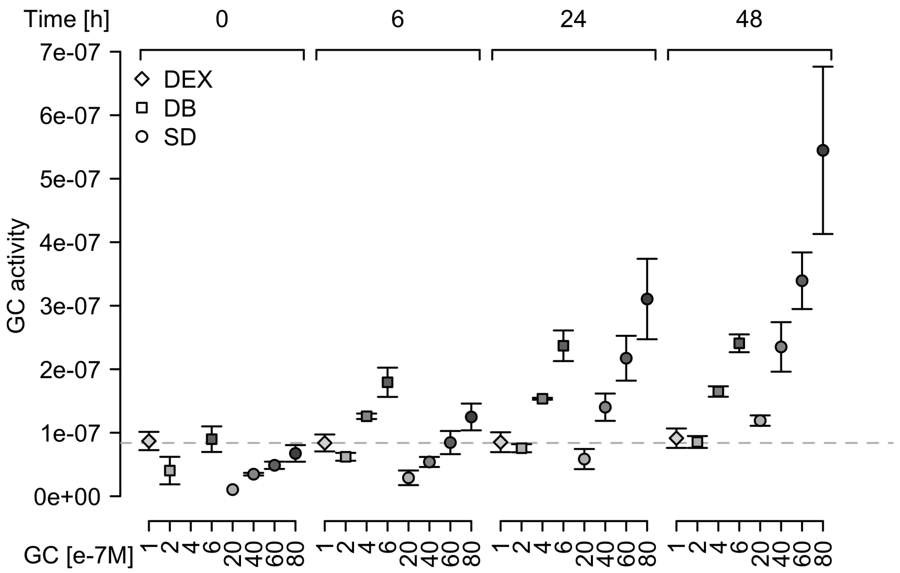

Supplementary Figure S1: Active GC-concentrations measured with the GC-bioactivity assay [1] in cell supernatants of dexamethasone (DEX, diamonds), Dexabene (DB, rectangles) and Solu-dacortin (SD, circles) treated C7H2 cells for the indicated time points and concentrations. Shown are mean and standard deviations from 3 independent experiments.

Our GC-bioactivity results reconfirmed the PRED:DEX dosage ratio of 7:1 that is being used in most clinical trials [2]. This dosage ratio was however only valid for the completely active form of the synthetic GCs (i.e. dexamethasone and prednisolone), as GC-bioactivity measurements of the water soluble variants Solu-dacortin [prednisolone 21-(sodium succinate)] and Dexabene [dexamethasone 21-(disodium phosphate)] yielded much higher differences in activity at early time points (60-fold higher concentration of Solu-dacortin resulted in a similar activity than 1.0e-7M DEX after 6 hours treatment while the difference in activity between DEX and Dexabene was 3-fold; supplementary Figure S1). In contrast to the active form of the synthetic GC Dexamethasone (DEX), which activity levels remain unchanged for the investigated time frame, both water soluble forms show increasing activity levels over treatment time (see Figure S1). Even more importantly, both water-soluble GCs showed different activity kinetics (supplementary Figure S1). The activation of these GC-variants might be much faster in vivo due to the higher availability of the enzymes required to activate the synthetic GC, thus, whether such differences would also be observed in vivo remains to be determined. The above described differences in activity kinetics renders these two water soluble GCs however incomparable in *in vitro* studies why we have opted to use the already active, ethanol soluble, dexamethasone and prednisolone instead. Also, the above described activation kinetics might explain the higher PRED:DEX ratios determined in other *in vitro* studies [3][4].

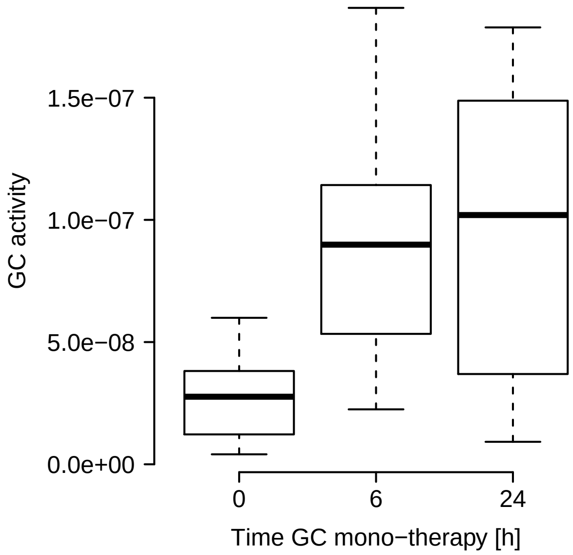

Supplementary Figure S2: Active GC levels (represented as dexamethasone equivalent concentration) measured in serum samples of 8 childhood B-ALL patients prior to and during systemic GC mono-therapy.

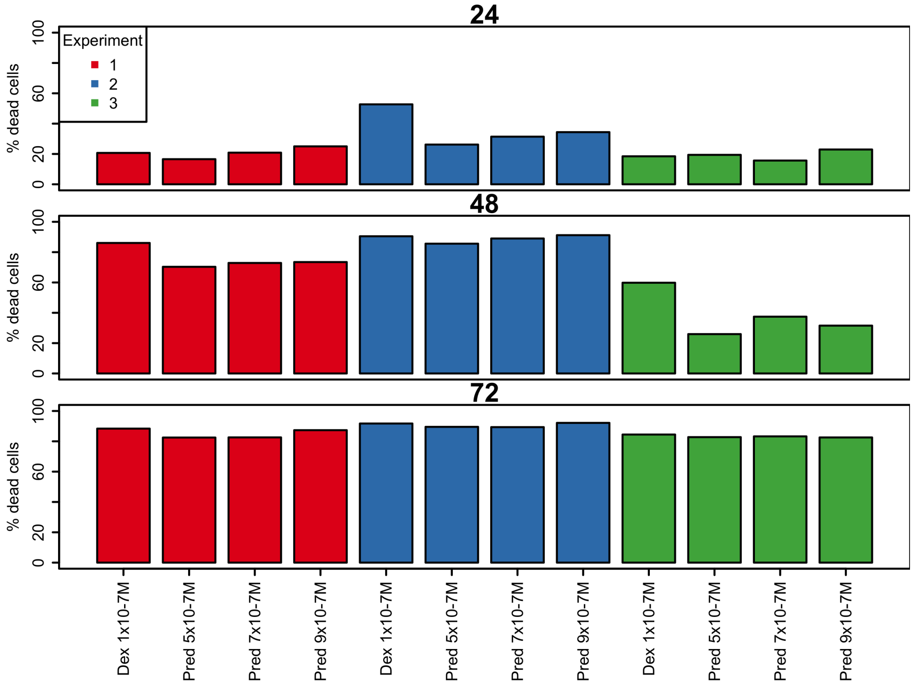

Supplementary Figure S3: Percentages of apoptotic and necrotic cells due to treatment with either dexamethasone (DEX) or prednisolone (PRED) in the indicated concentrations for each of the 3 experiments. Upper, middle and lower panel show the data after 24, 48 and 72 hours of treatment, respectively.

Comparison of transcriptional responses

Correlation of M-values

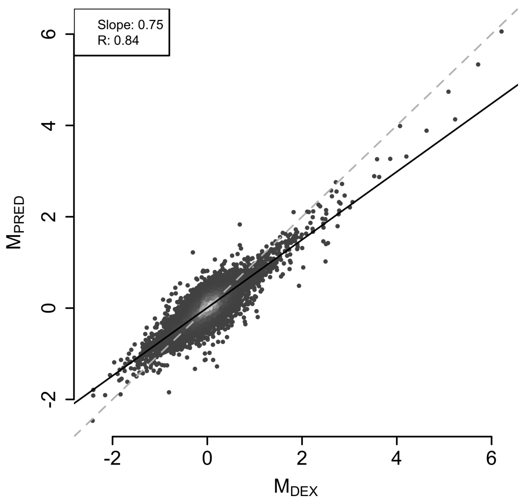

Supplementary Figure S4: Correlation of M-values (log2 fold change values) representing GC-regulation of the genes due to DEX (x-axis) or PRED (y-axis). The dashed grey line corresponds to the identity line, the black solid line the linear regression fit to the data cloud.

Comparison of GC-regulated genes

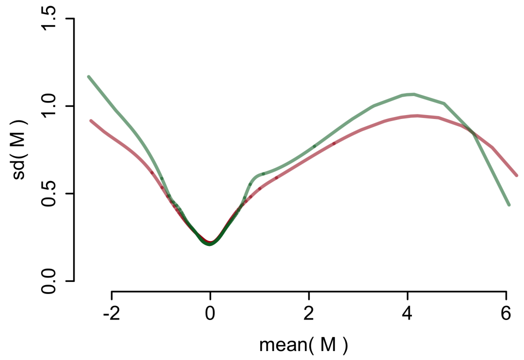

Supplementary Figure S5: Average gene regulations (x-axis) against variance of regulations (y-axis) for DEX (red line) and PRED (green line) treated samples (average and standard deviation across 3 replicates).

For both up- and down-regulated genes the inter-replicate variance is larger for the PRED treated samples (see Figure S5 above). This, in combination with the overall slightly lower extent of regulation (see Figure S4), results in lower significance levels and thus less significantly regulated genes identified for PRED treatment.

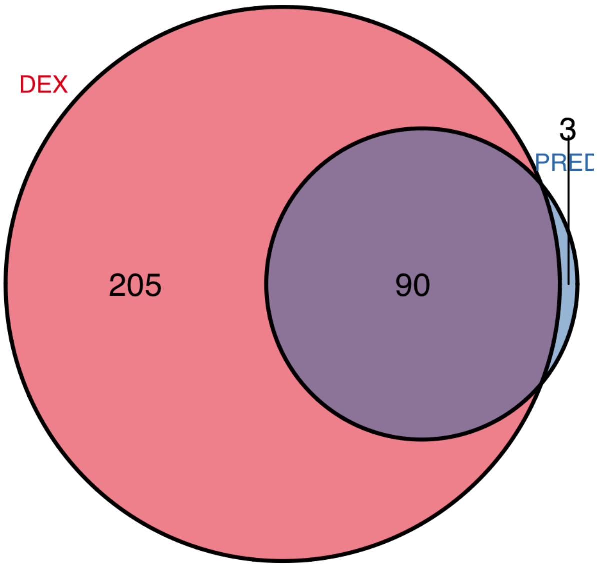

Supplementary Figure S6: Comparison of genes found significantly regulated by PRED or DEX treatment.

Comparison of genes found to be significantly regulated ($p_{BH} < 0.05$ and $|M| > 1$) by either PRED or DEX treatment.

All of the significant PRED-regulated genes were also significantly regulated by DEX, 3 of them however being slightly below the M-value cut-off in DEX treatment (highlighted blue in Table S1). Also, most of the genes found significantly regulated only by DEX were just below the p-value, M-value or both cut-offs. Specifically, of the 205 genes found significantly regulated only by DEX, 80 passed the M-value but not the p-value cut-off in PRED treated samples (highlighted green in Table S1) and 12 did not reach the regulation cut-off while passing the significance cut-off (highlighted blue in Table S1). Also, 92 of the remaining 113 genes were, with an M-value between 0.7 and 1, just below the cut-off for regulation in PRED treatment. The remaining 21 genes are shown in Table S3.

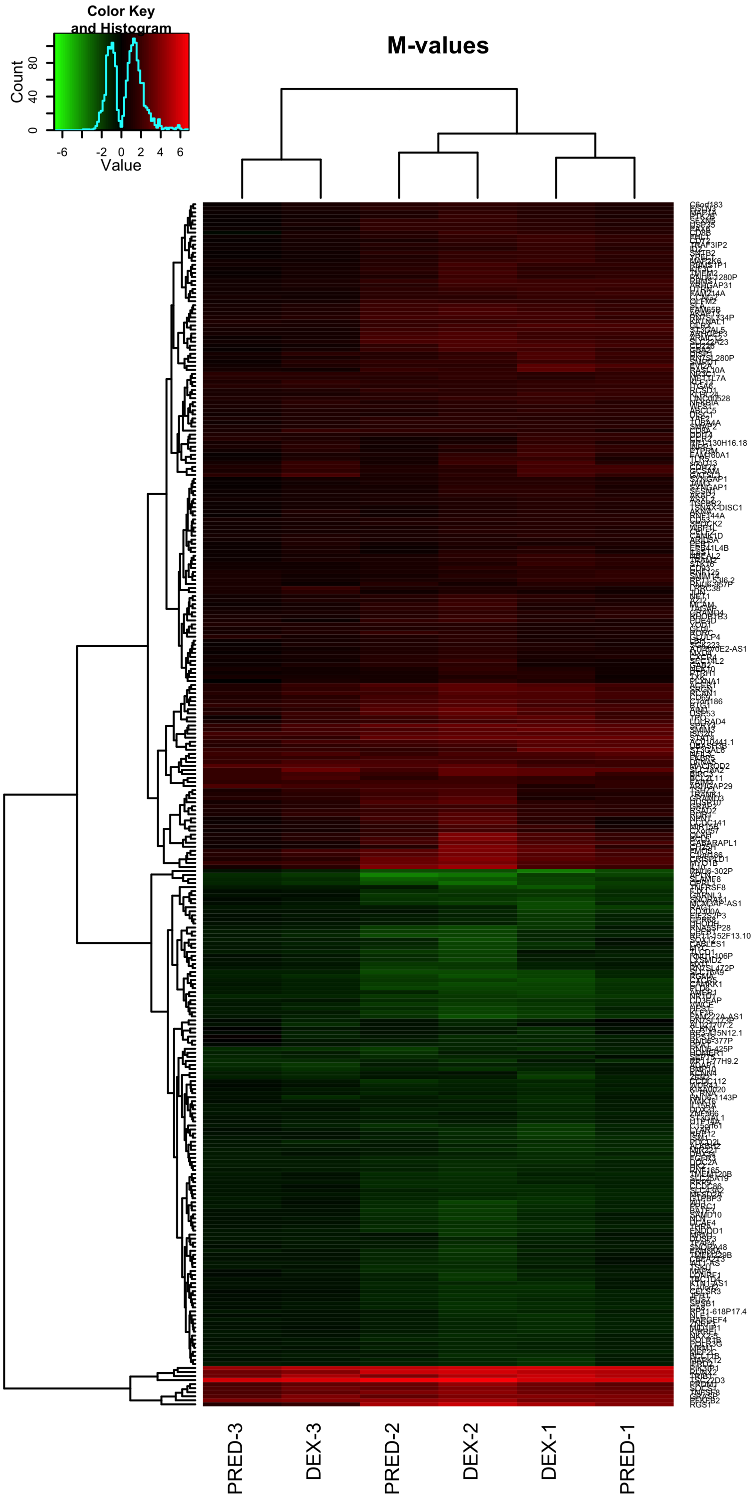

Supplementary Figure S7: Heatmap on the per-experiment M-values of genes significantly regulated by DEX and/or PRED.

Probe set ID	Gene name	No. probes	Gene biotype	$p_{BH}.D$	$M.D$	$p_{BH}.P$	$M.P$	$M.PvsD$	GBR
74ENST00000372382	TSC22D3	14	protein_coding	2.0e-05	6.21	2.4e-05	6.06	-0.15	YY
74ENST00000215912	PIK3IP1	23	protein_coding	4.2e-05	5.72	4.9e-05	5.34	-0.38	Y-
74ENST00000371432	RUNX2	18	protein_coding	7.2e-04	5.09	1.4e-03	4.74	-0.35	–
74ENST00000519576	TRIB1	10	protein_coding	1.7e-04	5.23	7.4e-04	4.13	-1.10	YY
74ENST00000411990	PFKFB2	14	protein_coding	4.2e-05	4.07	4.9e-05	3.99	-0.08	YY
74ENST00000546756	GRASP	15	protein_coding	1.7e-04	3.86	6.6e-04	3.27	-0.59	YY
74ENST00000223795	TNFSF8	30	protein_coding	3.9e-04	3.58	7.4e-04	3.26	-0.32	YY
74ENST00000332029	SOCS1	16	protein_coding	1.3e-04	3.52	6.0e-04	2.89	-0.63	YY
74ENST00000369096	PRDM1	30	protein_coding	3.3e-04	3.63	1.2e-03	2.87	-0.76	YY
74ENST00000392320	STAT4	24	protein_coding	6.1e-03	2.71	9.5e-03	2.76	0.04	–
74ENST00000560741	ISG20	9	protein_coding	3.3e-04	2.61	6.0e-04	2.57	-0.04	YY
74ENST00000392316	MYO1B	31	protein_coding	6.5e-03	2.73	1.6e-02	2.55	-0.18	–
74ENST00000427399	APLN	12	protein_coding	2.0e-02	-2.42	4.3e-02	-2.47	-0.05	YY
74ENST00000344120	SPRY4	29	protein_coding	1.7e-03	2.90	6.6e-03	2.46	-0.43	–
74ENST00000402914	MACROD2	21	protein_coding	4.2e-04	2.63	7.4e-04	2.45	-0.18	–
74ENST00000298472	SLC18A2	32	protein_coding	7.2e-04	3.07	5.2e-03	2.32	-0.74	–
74ENST00000526627	SMIM3	27	protein_coding	1.8e-03	2.78	8.0e-03	2.32	-0.46	
74ENST00000460774	ST3GAL6	13	protein_coding	1.1e-02	2.35	2.4e-02	2.26	-0.08	Y-
74ENST00000297689	NFIL3	25	protein_coding	7.2e-04	2.38	1.4e-03	2.23	-0.15	–
74ENST00000533174	FMO5	9	protein_coding	3.3e-03	3.01	2.1e-02	2.21	-0.80	–
74ENST00000600109	AC010441.1	23	protein_coding	1.7e-03	2.51	6.1e-03	2.15	-0.36	
74ENST00000256015	BTG1	22	protein_coding	1.7e-03	2.24	3.5e-03	2.12	-0.12	–
74ENST00000542713	FKBP5	17	protein_coding	5.4e-04	2.11	7.4e-04	2.11	0.00	YY
74ENST00000446822	DFNA5	10	protein_coding	1.4e-03	2.14	2.2e-03	2.10	-0.04	–
74ENST00000370217	ARHGAP29	13	protein_coding	9.7e-03	1.82	9.1e-03	2.10	0.28	–
74ENST00000535438	AIM1	15	protein_coding	1.1e-02	2.51	4.5e-02	2.07	-0.44	YY
74ENST00000603378	C1orf186	12	protein_coding	6.1e-03	2.77	4.2e-02	2.05	-0.72	
74ENST00000284273	UBASH3B	46	protein_coding	5.8e-03	2.23	1.6e-02	1.99	-0.24	–
74ENST00000301452	ACER1	31	protein_coding	3.9e-03	2.43	1.7e-02	1.96	-0.47	–
74ENST00000274030	USP53	36	protein_coding	4.1e-03	2.49	2.1e-02	1.95	-0.55	–
74ENST00000355631	OPRL1	24	protein_coding	7.3e-03	-2.41	3.8e-02	-1.91	0.49	–
74ENST00000368104	SLAMF8	15	protein_coding	1.2e-02	-2.16	4.2e-02	-1.91	0.25	Y-
74ENST00000228434	CD69	28	protein_coding	4.1e-03	2.18	1.4e-02	1.87	-0.31	YY
74ENST00000399272	RCAN1	14	protein_coding	9.9e-04	2.45	7.2e-03	1.85	-0.60	YY
74ENST00000263464	BIRC3	34	protein_coding	5.6e-04	2.75	8.0e-03	1.79	-0.96	YY
74ENST00000242465	SRGN	22	protein_coding	4.2e-03	2.45	3.3e-02	1.76	-0.69	–
74ENST00000331555	C1orf186	23	protein_coding	1.8e-03	2.22	9.5e-03	1.74	-0.49	
74ENST00000529560	FAIM3	10	protein_coding	5.4e-04	1.87	1.1e-03	1.72	-0.15	YY
74ENST00000380615	KATNAL1	33	protein_coding	1.1e-02	1.59	1.8e-02	1.65	0.05	Y-
74ENST00000586222	LDLRAD4	10	protein_coding	4.1e-03	2.09	2.1e-02	1.63	-0.45	YY
74ENST00000246672	NR1D1	30	protein_coding	3.0e-03	-1.69	6.1e-03	-1.62	0.07	–
74ENST00000405953	BCL2L11	15	protein_coding	9.5e-04	2.00	5.0e-03	1.61	-0.39	–
74ENST00000465067	RN7SL334P	16	misc_RNA	1.3e-02	1.84	4.3e-02	1.58	-0.25	–
74ENST00000321560	PLD6	30	protein_coding	1.1e-02	-1.83	4.3e-02	-1.56	0.26	–
74ENST00000416954	NR3C1	17	protein_coding	9.5e-04	1.54	1.2e-03	1.55	0.02	–
74ENST00000371080	ROR1	20	protein_coding	8.6e-03	1.97	4.3e-02	1.55	-0.43	–
74ENST00000292174	CXCR5	22	protein_coding	1.1e-02	-1.81	4.2e-02	-1.55	0.26	Y-
74ENST00000427060	SLA	23	protein_coding	1.1e-02	1.81	4.3e-02	1.53	-0.28	YY
74ENST00000383877	RNU6-425P	24	snRNA	1.4e-02	-1.50	2.7e-02	-1.50	-0.00	–
74ENST00000378023	FAM65B	18	protein_coding	9.7e-03	1.83	4.3e-02	1.49	-0.35	–
74ENST00000554387	RGMA	18	protein_coding	5.3e-03	-2.05	4.3e-02	-1.47	0.59	–
74ENST00000398788	CDH23	22	protein_coding	8.6e-03	1.83	4.2e-02	1.47	-0.36	–
74ENST00000537350	RCSD1	17	protein_coding	8.6e-03	1.44	1.5e-02	1.46	0.02	Y-
74ENST00000399090	GRAP2	15	protein_coding	4.9e-03	1.65	1.5e-02	1.46	-0.19	YY
74ENST00000307145	KLF13	28	protein_coding	3.3e-03	1.58	8.2e-03	1.44	-0.14	–
74ENST00000428977	TRANK1	25	protein_coding	1.1e-02	1.67	4.3e-02	1.41	-0.27	–
74ENST00000442250	ITGA6	28	protein_coding	1.7e-03	1.53	4.9e-03	1.41	-0.12	YY
74ENST00000548553	METTL7A	30	protein_coding	3.9e-03	1.47	8.0e-03	1.40	-0.07	YY
74ENST00000565934	RP11-77H9.2	6	antisense	2.8e-02	-1.28	4.7e-02	-1.38	-0.10	
74ENST00000250916	KLF16	27	protein_coding	1.1e-02	-1.74	4.9e-02	-1.38	0.37	–
74ENST00000509972	CCNG2	14	protein_coding	3.0e-02	1.25	4.9e-02	1.35	0.10	–
74ENST00000600723	LINC00528	24	lincRNA	1.7e-03	1.67	8.2e-03	1.35	-0.33	
74ENST00000450751	CPEB1	28	protein_coding	1.6e-02	-1.53	4.9e-02	-1.33	0.20	–
74ENST00000535710	VWCE	9	protein_coding	1.0e-02	-1.61	4.3e-02	-1.32	0.29	–
74ENST00000541728	RSAD2	17	protein_coding	3.9e-03	1.70	1.9e-02	1.32	-0.38	Y-
74ENST00000404953	GATSL3	22	protein_coding	3.9e-03	2.00	4.2e-02	1.32	-0.69	Y-
74ENST00000476808	KLHL24	18	protein_coding	9.7e-03	1.49	3.3e-02	1.31	-0.18	–
74ENST00000330258	AMER1	36	protein_coding	8.1e-03	-1.77	4.8e-02	-1.31	0.46	–
74ENST00000535690	HOMER1	13	protein_coding	2.5e-02	-0.91	1.5e-02	-1.25	-0.35	Y-
74ENST00000589804	CD3EAP	22	protein_coding	1.4e-02	-1.47	4.8e-02	-1.25	0.22	–
74ENST00000307365	DDIT4	30	protein_coding	5.8e-03	1.32	1.3e-02	1.25	-0.07	–
74ENST00000412135	FGFR3	21	protein_coding	6.1e-03	-1.19	8.3e-03	-1.24	-0.05	–
74ENST00000503569	WFS1	36	protein_coding	3.2e-03	1.42	9.5e-03	1.22	-0.20	YY
74ENST00000442791	YAF2	20	protein_coding	4.9e-03	1.29	1.1e-02	1.20	-0.10	YY
74ENST00000540377	TMEM120B	11	protein_coding	2.9e-02	-1.11	4.8e-02	-1.20	-0.09	–
74ENST00000372718	SMAP2	24	protein_coding	4.2e-03	1.25	8.4e-03	1.19	-0.06	Y-
74ENST00000408031	GRAMD4	10	protein_coding	1.5e-02	1.28	4.3e-02	1.18	-0.11	–

Probe set ID	Gene name	No. probes	Gene biotype	$p_{BH}.D$	$M.D$	$p_{BH}.P$	$M.P$	$M.PvsD$	GBR
74ENST00000217740	RNF125	26	protein_coding	2.4e-02	1.14	4.7e-02	1.18	0.03	Y-
74ENST00000409781	CD8A	14	protein_coding	5.3e-03	1.68	4.7e-02	1.15	-0.54	YY
74ENST00000232424	HES1	23	protein_coding	3.0e-03	-1.82	4.2e-02	-1.13	0.69	Y-
74ENST00000557389	NFKBIA	21	protein_coding	1.6e-03	1.53	1.0e-02	1.12	-0.41	–
74ENST00000564357	DOC2A	18	protein_coding	5.0e-03	-1.29	1.6e-02	-1.12	0.16	–
74ENST00000363652	RNU6-957P	7	snRNA	4.1e-02	0.86	4.2e-02	1.11	0.25	–
74ENST00000367084	YOD1	20	protein_coding	5.0e-03	1.10	8.3e-03	1.11	0.01	YY
74ENST00000295379	BMP10	28	protein_coding	3.3e-03	-1.44	1.8e-02	-1.10	0.35	–
74ENST00000324731	GLULP4	5	pseudogene	3.4e-02	0.95	4.8e-02	1.09	0.14	–
74ENST00000427120	ABCC5	12	protein_coding	5.0e-03	1.34	2.1e-02	1.08	-0.25	YY
74ENST00000420632	MFSD2A	29	protein_coding	2.0e-02	-1.13	4.7e-02	-1.08	0.05	–
74ENST00000232888	RRP9	38	protein_coding	1.5e-02	-1.11	3.6e-02	-1.07	0.04	–
74ENST00000427737	TUBA4A	11	protein_coding	1.0e-02	1.28	4.3e-02	1.06	-0.22	YY
74ENST00000453175	ADAP1	12	protein_coding	9.2e-03	-1.31	4.7e-02	-1.02	0.29	–
74ENST00000271324	CD53	24	protein_coding	7.7e-03	1.12	2.1e-02	1.01	-0.11	YY
74ENST00000339526	GLUL	14	protein_coding	8.6e-03	1.10	2.2e-02	1.00	-0.10	YY

Supplementary Table S1: Significantly differentially expressed genes between 6 hours PRED and EtHO treated cells. Values for DEX and PRED treatments are shown in columns with suffix D and P, respectively. Column $M.PvsD$ displays the difference in GC-regulation between PRED and DEX treatment. Column GBR contains ChIP-chip determined GR-DNA interaction information from [5] (with *YY* and *Y-* indicating an identified GR-DNA interaction in their promoter region of the gene with or without the presence of a GR-response element, respectively; – represents genes without a GR-DNA interaction and an empty cell is shown for genes that could not be detected by ChIP-chip. Not highlighted rows indicate genes significantly regulated due to PRED as well as to DEX exposure. Blue coloring highlights genes which don't meet the regulation, ($|M| > 1$) but satisfy the significance cut-off ($p_{BH} < 0.05$) in DEX treated samples.

Probe set ID	Gene name	No. probes	Gene biotype	$p_{BH}.D$	$M.D$	$p_{BH}.P$	$M.P$	$M.PvsD$	GBR
74ENST00000372382	TSC22D3	14	protein_coding	2.0e-05	6.21	2.4e-05	6.06	-0.15	YY
74ENST00000215912	PIK3IP1	23	protein_coding	4.2e-05	5.72	4.9e-05	5.34	-0.38	Y-
74ENST00000519576	TRIB1	10	protein_coding	1.7e-04	5.23	7.4e-04	4.13	-1.10	YY
74ENST00000371432	RUNX2	18	protein_coding	7.2e-04	5.09	1.4e-03	4.74	-0.35	–
74ENST00000469578	RGS1	13	protein_coding	2.1e-02	4.20	9.6e-02	3.32	-0.88	–
74ENST00000411990	PFKFB2	14	protein_coding	4.2e-05	4.07	4.9e-05	3.99	-0.08	YY
74ENST00000546756	GRASP	15	protein_coding	1.7e-04	3.86	6.6e-04	3.27	-0.59	YY
74ENST00000369096	PRDM1	30	protein_coding	3.3e-04	3.63	1.2e-03	2.87	-0.76	YY
74ENST00000223795	TNFSF8	30	protein_coding	3.9e-04	3.58	7.4e-04	3.26	-0.32	YY
74ENST00000332029	SOCS1	16	protein_coding	1.3e-04	3.52	6.0e-04	2.89	-0.63	YY
74ENST00000298472	SLC18A2	32	protein_coding	7.2e-04	3.07	5.2e-03	2.32	-0.74	–
74ENST00000533174	FMO5	9	protein_coding	3.3e-03	3.01	2.1e-02	2.21	-0.80	–
74ENST00000344120	SPRY4	29	protein_coding	1.7e-03	2.90	6.6e-03	2.46	-0.43	–
74ENST00000523524	CRISPLD1	19	protein_coding	1.9e-02	2.81	7.0e-02	2.29	-0.52	–
74ENST00000423557	IL10	32	protein_coding	4.1e-02	2.78	1.6e-01	2.13	-0.65	YY
74ENST00000526627	SMIM3	27	protein_coding	1.8e-03	2.78	8.0e-03	2.32	-0.46	
74ENST00000603378	C1orf186	12	protein_coding	6.1e-03	2.77	4.2e-02	2.05	-0.72	
74ENST00000263464	BIRC3	34	protein_coding	5.6e-04	2.75	8.0e-03	1.79	-0.96	YY
74ENST00000392316	MYO1B	31	protein_coding	6.5e-03	2.73	1.6e-02	2.55	-0.18	–
74ENST00000392320	STAT4	24	protein_coding	6.1e-03	2.71	9.5e-03	2.76	0.04	–
74ENST00000402914	MACROD2	21	protein_coding	4.2e-04	2.63	7.4e-04	2.45	-0.18	–
74ENST00000560741	ISG20	9	protein_coding	3.3e-04	2.61	6.0e-04	2.57	-0.04	YY
74ENST00000429028	OLAH	14	protein_coding	2.0e-02	2.55	1.8e-01	1.42	-1.12	–
74ENST00000535438	AIM1	15	protein_coding	1.1e-02	2.51	4.5e-02	2.07	-0.44	YY
74ENST00000600109	AC010441.1	23	protein_coding	1.7e-03	2.51	6.1e-03	2.15	-0.36	
74ENST00000450123	BCL6	20	protein_coding	2.0e-02	2.50	1.8e-01	1.40	-1.10	–
74ENST00000371852	CH25H	25	protein_coding	3.8e-02	2.50	3.9e-01	1.02	-1.47	YY
74ENST00000274030	USP53	36	protein_coding	4.1e-03	2.49	2.1e-02	1.95	-0.55	–
74ENST00000242465	SRGN	22	protein_coding	4.2e-03	2.45	3.3e-02	1.76	-0.69	–
74ENST00000399272	RCAN1	14	protein_coding	9.9e-04	2.45	7.2e-03	1.85	-0.60	YY
74ENST00000425083	TPO	13	protein_coding	9.2e-03	2.45	1.0e-01	1.46	-0.99	–
74ENST00000301452	ACER1	31	protein_coding	3.9e-03	2.43	1.7e-02	1.96	-0.47	–
74ENST00000427399	APLN	12	protein_coding	2.0e-02	-2.42	4.3e-02	-2.47	-0.05	YY
74ENST00000355631	OPRL1	24	protein_coding	7.3e-03	-2.41	3.8e-02	-1.91	0.49	–
74ENST00000365249	RNU6-302P	18	snRNA	3.6e-02	-2.41	1.5e-01	-1.79	0.62	–
74ENST00000297689	NFIL3	25	protein_coding	7.2e-04	2.38	1.4e-03	2.23	-0.15	–
74ENST00000460774	ST3GAL6	13	protein_coding	1.1e-02	2.35	2.4e-02	2.26	-0.08	Y-
74ENST00000256015	BTG1	22	protein_coding	1.7e-03	2.24	3.5e-03	2.12	-0.12	–
74ENST00000541960	GABARAPL1	11	protein_coding	3.5e-02	2.23	1.5e-01	1.67	-0.56	Y-
74ENST00000284273	UBASH3B	46	protein_coding	5.8e-03	2.23	1.6e-02	1.99	-0.24	–
74ENST00000331555	C1orf186	23	protein_coding	1.8e-03	2.22	9.5e-03	1.74	-0.49	
74ENST00000228434	CD69	28	protein_coding	4.1e-03	2.18	1.4e-02	1.87	-0.31	YY
74ENST00000368104	SLAMF8	15	protein_coding	1.2e-02	-2.16	4.2e-02	-1.91	0.25	Y-
74ENST00000446822	DFNA5	10	protein_coding	1.4e-03	2.14	2.2e-03	2.10	-0.04	–

Probe set ID	Gene name	No. probes	Gene biotype	$p_{BH}.D$	$M.D$	$p_{BH}.P$	$M.P$	$M.PvsD$	GBR
74ENST00000542713	FKBP5	17	protein_coding	5.4e-04	2.11	7.4e-04	2.11	0.00	YY
74ENST00000586222	LDLRAD4	10	protein_coding	4.1e-03	2.09	2.1e-02	1.63	-0.45	YY
74ENST00000554387	RGMA	18	protein_coding	5.3e-03	-2.05	4.3e-02	-1.47	0.59	–
74ENST00000459497	snoU13	25	snoRNA	7.0e-04	2.03	4.7e-02	0.89	-1.14	–
74ENST00000404953	GATSL3	22	protein_coding	3.9e-03	2.00	4.2e-02	1.32	-0.69	Y-
74ENST00000405953	BCL2L11	15	protein_coding	9.5e-04	2.00	5.0e-03	1.61	-0.39	–
74ENST00000371080	ROR1	20	protein_coding	8.6e-03	1.97	4.3e-02	1.55	-0.43	–
74ENST00000413146	TNFRSF8	18	protein_coding	2.3e-02	-1.96	1.7e-01	-1.19	0.78	–
74ENST00000577287	CD226	14	protein_coding	4.2e-02	1.90	1.7e-01	1.39	-0.51	–
74ENST00000284476	DISP1	36	protein_coding	1.1e-02	1.90	8.2e-02	1.29	-0.61	–
74ENST00000379979	GLRX	10	protein_coding	2.0e-02	1.90	5.9e-02	1.66	-0.23	–
74ENST00000394510	AKAP13	42	protein_coding	1.4e-02	1.89	5.1e-02	1.55	-0.34	–
74ENST00000348335	CAMKK1	23	protein_coding	1.5e-02	-1.89	7.0e-02	-1.44	0.45	–
74ENST00000467177	SLC22A23	9	protein_coding	2.4e-02	1.88	7.7e-02	1.64	-0.24	Y-
74ENST00000529560	FAIM3	10	protein_coding	5.4e-04	1.87	1.1e-03	1.72	-0.15	YY
74ENST00000395348	SLC16A9	29	protein_coding	2.0e-02	-1.86	7.0e-02	-1.58	0.27	–
74ENST00000366899	DUSP10	27	protein_coding	3.4e-02	1.85	1.3e-01	1.47	-0.38	Y-
74ENST00000541723	FAM222A-AS1	18	antisense	1.1e-02	-1.85	6.6e-02	-1.35	0.50	
74ENST00000465067	RN7SL334P	16	misc_RNA	1.3e-02	1.84	4.3e-02	1.58	-0.25	–
74ENST00000378023	FAM65B	18	protein_coding	9.7e-03	1.83	4.3e-02	1.49	-0.35	–
74ENST00000398788	CDH23	22	protein_coding	8.6e-03	1.83	4.2e-02	1.47	-0.36	–
74ENST00000321560	PLD6	30	protein_coding	1.1e-02	-1.83	4.3e-02	-1.56	0.26	–
74ENST00000232424	HES1	23	protein_coding	3.0e-03	-1.82	4.2e-02	-1.13	0.69	Y-
74ENST00000370217	ARHGAP29	13	protein_coding	9.7e-03	1.82	9.1e-03	2.10	0.28	–
74ENST00000462804	EVI2A	22	protein_coding	4.0e-02	1.81	1.3e-01	1.53	-0.28	Y-
74ENST00000292174	CXCR5	22	protein_coding	1.1e-02	-1.81	4.2e-02	-1.55	0.26	Y-
74ENST00000427060	SLA	23	protein_coding	1.1e-02	1.81	4.3e-02	1.53	-0.28	YY
74ENST00000240587	TSHZ3	21	protein_coding	1.4e-02	1.77	5.1e-02	1.47	-0.30	–
74ENST00000330258	AMER1	36	protein_coding	8.1e-03	-1.77	4.8e-02	-1.31	0.46	–
74ENST00000488580	GCSAM	11	protein_coding	1.5e-02	1.77	5.8e-02	1.43	-0.34	–
74ENST00000385045	MIR15B	25	miRNA	1.7e-02	1.76	1.6e-01	1.00	-0.76	–
74ENST00000496490	RN7SL280P	22	misc_RNA	2.0e-02	1.75	1.3e-01	1.18	-0.57	–
74ENST00000495373	ARHGEF3	19	protein_coding	3.7e-02	1.75	9.9e-02	1.61	-0.13	Y-
74ENST00000504304	NPNT	10	protein_coding	4.0e-02	1.75	2.3e-01	1.04	-0.71	–
74ENST00000250916	KLF16	27	protein_coding	1.1e-02	-1.74	4.9e-02	-1.38	0.37	–
74ENST00000420890	CCDC141	43	protein_coding	4.7e-02	1.74	3.4e-01	0.85	-0.88	–
74ENST00000373866	ARMC12	23	protein_coding	4.4e-02	1.73	1.3e-01	1.49	-0.24	YY
74ENST00000545786	GBA2	23	protein_coding	3.5e-02	1.73	1.5e-01	1.28	-0.45	YY
74ENST00000216101	RASL10A	19	protein_coding	4.7e-02	1.72	1.4e-01	1.47	-0.24	–
74ENST00000506445	GRAMD3	15	protein_coding	3.5e-02	1.71	9.6e-02	1.57	-0.13	–
74ENST00000541728	RSAD2	17	protein_coding	3.9e-03	1.70	1.9e-02	1.32	-0.38	Y-
74ENST00000246672	NR1D1	30	protein_coding	3.0e-03	-1.69	6.1e-03	-1.62	0.07	–
74ENST00000444998	MCM3AP-AS1	12	antisense	2.5e-02	-1.69	1.5e-01	-1.11	0.57	YY
74ENST00000409781	CD8A	14	protein_coding	5.3e-03	1.68	4.7e-02	1.15	-0.54	YY
74ENST00000428977	TRANK1	25	protein_coding	1.1e-02	1.67	4.3e-02	1.41	-0.27	–
74ENST00000600723	LINC00528	24	lincRNA	1.7e-03	1.67	8.2e-03	1.35	-0.33	
74ENST00000392753	RBMS1	10	protein_coding	2.3e-02	1.67	8.3e-02	1.41	-0.26	Y-
74ENST00000399090	GRAP2	15	protein_coding	4.9e-03	1.65	1.5e-02	1.46	-0.19	YY
74ENST00000393808	ST3GAL5	20	protein_coding	2.8e-02	1.65	6.0e-02	1.62	-0.03	–
74ENST00000482743	ARHGAP31	10	protein_coding	2.1e-02	1.65	1.1e-01	1.24	-0.41	Y-
74ENST00000363841	RNA5SP28	24	rRNA	3.1e-02	-1.65	1.4e-01	-1.19	0.45	–
74ENST00000526280	SMPD1	12	protein_coding	2.1e-02	1.64	1.1e-01	1.25	-0.39	–
74ENST00000322522	INPP1	25	protein_coding	7.7e-03	1.62	8.6e-02	0.98	-0.64	YY
74ENST00000535710	VWCE	9	protein_coding	1.0e-02	-1.61	4.3e-02	-1.32	0.29	–
74ENST00000400473	CABLES1	18	protein_coding	9.7e-03	-1.60	1.2e-01	-0.91	0.69	–
74ENST00000435205	FAM160A1	8	protein_coding	1.5e-02	1.59	2.0e-01	0.74	-0.85	–
74ENST00000380615	KATNAL1	33	protein_coding	1.1e-02	1.59	1.8e-02	1.65	0.05	Y-
74ENST00000307145	KLF13	28	protein_coding	3.3e-03	1.58	8.2e-03	1.44	-0.14	–
74ENST00000562833	RP11-152F13.10	17	protein_coding	2.8e-02	-1.56	7.1e-02	-1.46	0.10	
74ENST00000540964	TLR5	24	protein_coding	6.1e-03	1.56	1.5e-01	0.69	-0.86	–
74ENST00000377066	TMEM2	24	protein_coding	3.8e-02	1.56	1.8e-01	1.06	-0.50	–
74ENST00000259523	MYC	14	protein_coding	1.5e-02	-1.54	9.6e-02	-1.09	0.46	–
74ENST00000416954	NR3C1	17	protein_coding	9.5e-04	1.54	1.2e-03	1.55	0.02	–
74ENST00000450751	CPEB1	28	protein_coding	1.6e-02	-1.53	4.9e-02	-1.33	0.20	–
74ENST00000557389	NFKBIA	21	protein_coding	1.6e-03	1.53	1.0e-02	1.12	-0.41	–
74ENST00000544632	SOX12	12	protein_coding	2.5e-02	-1.53	2.2e-01	-0.80	0.74	–
74ENST00000442250	ITGA6	28	protein_coding	1.7e-03	1.53	4.9e-03	1.41	-0.12	YY
74ENST00000384151	SNORA51	25	snoRNA	4.3e-02	-1.52	1.8e-01	-1.11	0.41	–
74ENST00000367545	UTRN	78	protein_coding	2.8e-02	1.50	9.6e-02	1.28	-0.22	–
74ENST00000383877	RNU6-425P	24	snRNA	1.4e-02	-1.50	2.7e-02	-1.50	-0.00	–
74ENST00000458357	FHL1	9	protein_coding	2.1e-02	1.49	1.2e-01	1.08	-0.41	–
74ENST00000476808	KLHL24	18	protein_coding	9.7e-03	1.49	3.3e-02	1.31	-0.18	–
74ENST00000417216	RBMS1P1	7	pseudogene	2.9e-02	1.47	1.1e-01	1.19	-0.28	–
74ENST00000589804	CD3EAP	22	protein_coding	1.4e-02	-1.47	4.8e-02	-1.25	0.22	–
74ENST00000372548	CXorf57	42	protein_coding	2.8e-02	1.47	1.3e-01	1.06	-0.41	–
74ENST00000393759	CD8B	10	protein_coding	3.5e-02	1.47	2.3e-01	0.83	-0.64	–
74ENST00000548553	METTL7A	30	protein_coding	3.9e-03	1.47	8.0e-03	1.40	-0.07	YY

Probe set ID	Gene name	No. probes	Gene biotype	$p_{BH}.D$	$M.D$	$p_{BH}.P$	$M.P$	$M.PvsD$	GBR
74ENST00000560041	C15orf61	13	protein_coding	2.0e-02	-1.46	8.2e-02	-1.20	0.27	–
74ENST00000378430	CD72	12	protein_coding	2.4e-02	1.46	1.8e-01	0.89	-0.58	YY
74ENST00000365211	RNU6-1280P	25	snRNA	3.7e-02	1.46	1.8e-01	1.01	-0.45	–
74ENST00000589295	MAP2K6	11	protein_coding	2.9e-02	1.46	1.4e-01	1.06	-0.40	–
74ENST00000444013	PTPRM	28	protein_coding	1.8e-02	1.46	1.2e-01	0.99	-0.46	–
74ENST00000295379	BMP10	28	protein_coding	3.3e-03	-1.44	1.8e-02	-1.10	0.35	–
74ENST00000299440	RAG1	16	protein_coding	3.4e-02	-1.44	1.2e-01	-1.16	0.28	–
74ENST00000537350	RCSD1	17	protein_coding	8.6e-03	1.44	1.5e-02	1.46	0.02	Y-
74ENST00000503569	WFS1	36	protein_coding	3.2e-03	1.42	9.5e-03	1.22	-0.20	YY
74ENST00000449083	NET1	11	protein_coding	8.7e-03	1.42	7.0e-02	0.95	-0.47	–
74ENST00000317811	FJX1	26	protein_coding	2.8e-02	-1.42	1.2e-01	-1.10	0.32	–
74ENST00000527775	WT1	10	protein_coding	4.5e-02	-1.42	1.5e-01	-1.13	0.29	–
74ENST00000392861	ZEB2	15	protein_coding	1.6e-02	-1.41	9.6e-02	-1.00	0.41	–
74ENST00000405914	KIF3C	29	protein_coding	2.6e-02	1.40	1.2e-01	1.08	-0.32	–
74ENST00000571655	SNTB2	15	protein_coding	4.5e-02	1.38	1.7e-01	1.03	-0.35	
74ENST00000479665	AZI2	24	protein_coding	7.3e-03	1.38	7.0e-02	0.88	-0.50	–
74ENST00000587065	PDCD2L	10	protein_coding	4.1e-03	-1.37	3.2e-02	-0.98	0.39	YY
74ENST00000376085	LRRC38	21	protein_coding	1.1e-02	1.37	9.6e-02	0.87	-0.50	–
74ENST00000516125	RNU6-1143P	24	snRNA	3.3e-03	-1.37	5.2e-02	-0.78	0.59	–
74ENST00000243440	BATF3	21	protein_coding	2.9e-02	-1.37	1.1e-01	-1.11	0.25	–
74ENST00000339468	YPEL1	26	protein_coding	3.5e-02	1.37	1.7e-01	0.94	-0.43	–
74ENST00000560491	LYSMD2	9	protein_coding	3.6e-02	-1.35	1.8e-01	-0.93	0.42	–
74ENST00000399202	FAM214A	26	protein_coding	4.8e-02	1.35	1.2e-01	1.29	-0.06	–
74ENST00000410065	SFXN5	22	protein_coding	3.4e-02	1.35	1.5e-01	1.00	-0.35	–
74ENST00000278505	ENDOD1	25	protein_coding	1.4e-02	-1.34	6.2e-02	-1.04	0.31	–
74ENST00000359831	TRAF3IP2	30	protein_coding	2.5e-02	1.34	1.1e-01	1.05	-0.30	–
74ENST00000528910	USP35	14	protein_coding	2.6e-02	1.34	1.4e-01	0.93	-0.41	–
74ENST00000427120	ABCC5	12	protein_coding	5.0e-03	1.34	2.1e-02	1.08	-0.25	YY
74ENST00000590841	OLFM2	21	protein_coding	2.6e-02	1.34	6.6e-02	1.25	-0.09	–
74ENST00000502464	NLN	30	protein_coding	2.1e-02	-1.33	1.2e-01	-0.94	0.39	–
74ENST00000539444	DISC1	32	protein_coding	1.0e-02	1.33	6.2e-02	0.96	-0.38	YY
74ENST00000443829	NBEAL2	27	protein_coding	1.8e-02	1.33	1.1e-01	0.92	-0.41	–
74ENST00000439286	GARNL3	9	protein_coding	4.7e-02	-1.33	1.2e-01	-1.22	0.10	–
74ENST00000307365	DDIT4	30	protein_coding	5.8e-03	1.32	1.3e-02	1.25	-0.07	–
74ENST00000453496	C6orf183	21	polymorphic_pseudogene	1.8e-02	1.32	1.2e-01	0.89	-0.43	–
74ENST00000361933	CD300A	10	protein_coding	3.4e-02	-1.31	1.8e-01	-0.84	0.47	Y-
74ENST00000453175	ADAP1	12	protein_coding	9.2e-03	-1.31	4.7e-02	-1.02	0.29	–
74ENST00000489474	RPS18	4	protein_coding	3.6e-02	-1.31	3.6e-01	-0.57	0.74	
74ENST00000385135	MIR221	25	miRNA	1.8e-02	-1.30	7.3e-02	-1.03	0.27	–
74ENST00000452476	LYAR	26	protein_coding	2.4e-02	-1.30	1.2e-01	-0.98	0.32	–
74ENST00000442791	YAF2	20	protein_coding	4.9e-03	1.29	1.1e-02	1.20	-0.10	YY
74ENST00000182527	TRAM2	24	protein_coding	1.6e-02	1.29	8.6e-02	0.95	-0.34	–
74ENST00000546243	THRA	23	protein_coding	1.5e-02	-1.29	5.9e-02	-1.05	0.25	–
74ENST00000580518	TLCD1	17	protein_coding	4.9e-02	-1.29	1.3e-01	-1.16	0.13	–
74ENST00000434291	RP1-130H16.18	31	protein_coding	5.0e-03	1.29	4.8e-02	0.85	-0.44	Y-
74ENST00000564357	DOC2A	18	protein_coding	5.0e-03	-1.29	1.6e-02	-1.12	0.16	–
74ENST00000311916	C10orf2	22	protein_coding	3.6e-02	-1.29	1.8e-01	-0.87	0.42	–
74ENST00000408031	GRAMD4	10	protein_coding	1.5e-02	1.28	4.3e-02	1.18	-0.11	–
74ENST00000427737	TUBA4A	11	protein_coding	1.0e-02	1.28	4.3e-02	1.06	-0.22	YY
74ENST00000331129	ID2	9	protein_coding	2.5e-02	1.28	1.2e-01	0.94	-0.34	YY
74ENST00000536831	RRP12	34	protein_coding	3.3e-02	-1.28	1.3e-01	-0.98	0.30	–
74ENST00000565934	RP11-77H9.2	6	antisense	2.8e-02	-1.28	4.7e-02	-1.38	-0.10	
74ENST00000445132	CCR2	26	protein_coding	2.6e-02	1.27	8.3e-02	1.11	-0.16	YY
74ENST00000369886	SAMD10	18	protein_coding	3.0e-02	-1.27	1.4e-01	-0.92	0.34	–
74ENST00000509972	CCNG2	14	protein_coding	3.0e-02	1.25	4.9e-02	1.35	0.10	–
74ENST00000372718	SMAP2	24	protein_coding	4.2e-03	1.25	8.4e-03	1.19	-0.06	Y-
74ENST00000370012	PPRC1	15	protein_coding	3.6e-02	-1.25	9.6e-02	-1.16	0.09	–
74ENST00000357898	SPSB1	15	protein_coding	1.6e-02	-1.25	9.6e-02	-0.88	0.36	–
74ENST00000264036	MCAM	42	protein_coding	3.5e-02	1.25	1.4e-01	0.96	-0.28	–
74ENST00000342232	JPH1	27	protein_coding	2.8e-02	-1.24	1.4e-01	-0.87	0.37	–
74ENST00000543444	ALKBH2	16	protein_coding	9.7e-03	-1.24	5.8e-02	-0.90	0.34	–
74ENST00000394234	DCAF4	26	protein_coding	2.0e-02	-1.24	9.6e-02	-0.97	0.27	–
74ENST00000492799	RN7SL472P	25	misc_RNA	3.4e-02	-1.23	8.3e-02	-1.18	0.05	–
74ENST00000588679	RNF165	20	protein_coding	6.8e-03	-1.23	3.7e-02	-0.97	0.27	–
74ENST00000346049	PTK2B	35	protein_coding	2.9e-02	1.23	1.9e-01	0.73	-0.50	–
74ENST00000504179	RHOBTB3	10	protein_coding	3.3e-02	1.23	7.9e-02	1.18	-0.05	Y-
74ENST00000384382	RNU1-106P	22	snRNA	4.7e-02	-1.23	1.4e-01	-1.03	0.20	–
74ENST00000581301	MRM1	7	protein_coding	3.2e-02	-1.23	2.5e-01	-0.65	0.58	
74ENST00000317995	CA8	20	protein_coding	1.7e-02	-1.23	7.0e-02	-0.98	0.25	–
74ENST00000428356	EIF2S2P3	17	pseudogene	2.6e-02	-1.23	1.8e-01	-0.73	0.50	–
74ENST00000553215	EGLN3	9	protein_coding	2.2e-02	1.22	1.2e-01	0.87	-0.35	–
74ENST00000535815	GPR68	28	protein_coding	2.9e-02	-1.22	1.6e-01	-0.82	0.40	–
74ENST00000358923	PDE4D	14	protein_coding	4.9e-02	1.21	1.2e-01	1.16	-0.05	–
74ENST00000357195	BCL11B	22	protein_coding	7.3e-03	-1.21	5.8e-02	-0.82	0.39	–
74ENST00000458008	FAM86A	9	protein_coding	3.4e-02	-1.21	1.4e-01	-0.91	0.30	–
74ENST00000572887	DHODH	24	protein_coding	2.1e-02	-1.21	1.1e-01	-0.90	0.31	–
74ENST00000395780	MAPK12	23	protein_coding	2.1e-02	-1.20	1.1e-01	-0.91	0.29	–

Probe set ID	Gene name	No. probes	Gene biotype	$p_{BH}.D$	$M.D$	$p_{BH}.P$	$M.P$	$M.PvsD$	GBR
74ENST00000290573	HK2	5	protein_coding	3.9e-03	-1.20	1.6e-02	-0.98	0.22	–
74ENST00000426499	IFRD2	9	protein_coding	1.8e-02	-1.20	7.3e-02	-0.95	0.25	–
74ENST00000262487	ISM1	28	protein_coding	3.7e-02	-1.19	1.1e-01	-1.09	0.11	YY
74ENST00000378845	CAMK1D	22	protein_coding	5.0e-03	1.19	2.1e-02	0.96	-0.22	–
74ENST00000382031	MAP1A	37	protein_coding	2.2e-02	1.19	1.2e-01	0.87	-0.31	–
74ENST00000412135	FGFR3	21	protein_coding	6.1e-03	-1.19	8.3e-03	-1.24	-0.05	–
74ENST00000395109	SEPT5	14	protein_coding	3.5e-02	-1.18	1.1e-01	-1.03	0.15	–
74ENST00000436639	SESN1	28	protein_coding	2.0e-02	1.18	1.4e-01	0.75	-0.43	Y-
74ENST00000425511	TBC1D4	29	protein_coding	2.4e-02	-1.18	1.4e-01	-0.80	0.38	–
74ENST00000369889	WBP1L	18	protein_coding	6.1e-03	1.18	4.8e-02	0.82	-0.36	YY
74ENST00000442296	MXI1	9	protein_coding	4.8e-02	-1.17	1.2e-01	-1.06	0.11	Y-
74ENST00000409743	STK16	24	protein_coding	3.2e-02	1.17	1.2e-01	0.96	-0.21	YY
74ENST00000600909	KCNN4	12	protein_coding	3.8e-02	-1.17	1.9e-01	-0.77	0.41	–
74ENST00000428982	SYNGAP1	22	protein_coding	1.6e-02	1.17	1.8e-01	0.61	-0.56	–
74ENST00000434267	MAP4	27	protein_coding	4.0e-02	-1.17	2.0e-01	-0.76	0.40	Y-
74ENST00000246006	CD93	32	protein_coding	2.0e-02	1.17	8.4e-02	0.93	-0.24	–
74ENST00000361619	GTPBP3	24	protein_coding	2.7e-02	-1.17	5.9e-02	-1.15	0.02	–
74ENST00000434623	AKAP2	9	protein_coding	1.7e-02	1.17	1.2e-01	0.76	-0.40	–
74ENST00000356728	RORC	28	protein_coding	2.9e-02	1.16	6.3e-02	1.13	-0.03	Y-
74ENST00000223791	AKNA	29	protein_coding	1.5e-02	1.16	1.3e-01	0.70	-0.46	Y-
74ENST00000588019	NLE1	12	protein_coding	2.9e-02	-1.16	1.7e-01	-0.76	0.40	–
74ENST00000393409	PLXNA1	36	protein_coding	1.3e-02	1.15	3.0e-01	0.41	-0.75	–
74ENST00000505729	SMIM14	10	protein_coding	3.8e-02	1.15	9.1e-02	1.11	-0.04	–
74ENST00000301335	SLC43A2	27	protein_coding	2.3e-02	-1.15	6.9e-02	-1.02	0.12	–
74ENST00000367066	TAGAP	43	protein_coding	3.4e-02	1.15	9.6e-02	1.04	-0.10	Y-
74ENST00000217740	RNF125	26	protein_coding	2.4e-02	1.14	4.7e-02	1.18	0.03	Y-
74ENST00000524526	LONRF1	9	protein_coding	4.8e-02	-1.14	2.2e-01	-0.75	0.39	–
74ENST00000379109	PAX6	11	protein_coding	4.6e-02	1.14	1.2e-01	1.03	-0.11	–
74ENST00000539331	RAPGEF4	27	protein_coding	2.2e-02	-1.14	9.6e-02	-0.90	0.23	–
74ENST00000340208	MEF2C	16	protein_coding	9.2e-03	-1.14	5.2e-02	-0.83	0.30	YY
74ENST00000515965	RNU6-377P	24	snRNA	1.9e-02	-1.13	1.9e-01	-0.59	0.55	–
74ENST00000580151	SLC25A19	14	protein_coding	2.0e-02	-1.13	5.4e-02	-1.03	0.11	–
74ENST00000420632	MFSD2A	29	protein_coding	2.0e-02	-1.13	4.7e-02	-1.08	0.05	–
74ENST00000574215	NEK10	15	protein_coding	4.5e-02	1.12	2.3e-01	0.70	-0.42	–
74ENST00000271324	CD53	24	protein_coding	7.7e-03	1.12	2.1e-02	1.01	-0.11	YY
74ENST00000391143	SNORA48	25	snoRNA	2.3e-02	-1.12	1.7e-01	-0.69	0.43	–
74ENST00000357485	ARID5A	30	protein_coding	6.9e-03	1.12	4.3e-02	0.83	-0.29	–
74ENST00000379611	CCDC112	31	protein_coding	3.6e-02	-1.12	9.6e-02	-1.05	0.07	–
74ENST00000416587	RNF144A	9	protein_coding	2.6e-02	1.11	2.0e-01	0.62	-0.49	–
74ENST00000232888	RRP9	38	protein_coding	1.5e-02	-1.11	3.6e-02	-1.07	0.04	–
74ENST00000540377	TMEM120B	11	protein_coding	2.9e-02	-1.11	4.8e-02	-1.20	-0.09	–
74ENST00000461853	RN7SL173P	25	misc_RNA	9.1e-03	-1.11	5.1e-01	-0.23	0.88	–
74ENST00000592740	RP11-618P17.4	10	protein_coding	3.6e-02	-1.10	1.8e-01	-0.75	0.36	
74ENST00000164024	CELSR3	46	protein_coding	4.1e-02	-1.10	1.8e-01	-0.80	0.31	–
74ENST00000339526	GLUL	14	protein_coding	8.6e-03	1.10	2.2e-02	1.00	-0.10	YY
74ENST00000367084	YOD1	20	protein_coding	5.0e-03	1.10	8.3e-03	1.11	0.01	YY
74ENST00000335142	KTN1-AS1	23	antisense	3.8e-02	-1.10	1.6e-01	-0.81	0.28	YY
74ENST00000327483	CBFA2T3	36	protein_coding	3.4e-02	-1.09	1.8e-01	-0.73	0.36	–
74ENST00000381293	IL6ST	13	protein_coding	7.7e-03	1.09	1.7e-01	0.48	-0.61	–
74ENST00000543175	PTRH1	18	protein_coding	2.1e-02	1.09	2.0e-01	0.58	-0.51	–
74ENST00000544604	ZNRF3	32	protein_coding	2.1e-02	-1.09	9.2e-02	-0.86	0.23	–
74ENST00000354440	CELF2	23	protein_coding	8.1e-03	1.09	4.3e-02	0.85	-0.24	YY
74ENST00000371222	JUN	32	protein_coding	9.7e-03	1.09	1.1e-01	0.65	-0.44	–
74ENST00000409894	POLR1B	30	protein_coding	2.0e-02	-1.08	8.3e-02	-0.86	0.22	–
74ENST00000397885	KIAA0020	33	protein_coding	2.0e-02	-1.08	8.0e-02	-0.88	0.21	–
74ENST00000403484	SEC14L2	28	protein_coding	4.6e-02	1.08	2.1e-01	0.71	-0.37	–
74ENST00000330777	SGK223	30	protein_coding	1.6e-02	1.08	1.2e-01	0.67	-0.41	–
74ENST00000204517	TFAP4	26	protein_coding	1.3e-02	-1.08	9.6e-02	-0.72	0.36	–
74ENST00000461019	ATP6V0E2-AS1	27	antisense	2.0e-02	1.08	1.2e-01	0.74	-0.34	YY
74ENST00000328771	DHX34	25	protein_coding	2.6e-02	-1.07	6.3e-02	-1.02	0.06	–
74ENST00000583184	RP11-53I6.2	15	processed_transcript	3.7e-02	1.07	6.3e-02	1.15	0.08	
74ENST00000544928	AL021707.2	3	pseudogene	2.9e-02	-1.07	3.7e-01	-0.43	0.65	
74ENST00000227520	CCDC86	15	protein_coding	3.7e-02	-1.07	8.5e-02	-1.05	0.02	–
74ENST00000395071	SYNGAP1	27	protein_coding	2.0e-02	1.07	1.5e-01	0.66	-0.41	
74ENST00000356362	PUS7	45	protein_coding	2.7e-02	-1.07	8.5e-02	-0.93	0.14	–
74ENST00000517440	NKX2-5	9	protein_coding	2.9e-02	-1.07	1.1e-01	-0.89	0.18	–
74ENST00000397246	IL15RA	10	protein_coding	1.3e-02	-1.07	6.6e-02	-0.80	0.27	–
74ENST00000365352	Y_RNA	24	misc_RNA	2.1e-02	-1.06	4.2e-01	-0.34	0.72	–
74ENST00000337190	MXD4	24	protein_coding	3.6e-02	1.06	1.7e-01	0.76	-0.30	–
74ENST00000464412	LBH	11	protein_coding	2.0e-02	1.06	1.3e-01	0.70	-0.36	–
74ENST00000374557	EPB41L4B	21	protein_coding	1.4e-02	1.06	1.5e-01	0.58	-0.48	–
74ENST00000494911	WT1-AS	12	antisense	4.1e-02	-1.06	1.6e-01	-0.82	0.24	–
74ENST00000264220	PPAT	17	protein_coding	2.0e-02	-1.06	1.2e-01	-0.74	0.31	–
74ENST00000404926	RP3-415N12.1	14	pseudogene	4.1e-02	-1.05	5.0e-01	-0.35	0.71	–
74ENST00000416935	KIRREL	26	protein_coding	3.0e-02	-1.05	1.1e-01	-0.89	0.16	–
74ENST00000333090	TSKU	21	protein_coding	3.0e-02	-1.05	9.6e-02	-0.92	0.13	YY
74ENST00000602634	TSNAX-DISC1	49	protein_coding	1.6e-02	1.05	1.2e-01	0.69	-0.36	

Probe set ID	Gene name	No. probes	Gene biotype	$p_{BH}.D$	$M.D$	$p_{BH}.P$	$M.P$	$M.PvsD$	GBR
74ENST00000493391	ZNF566	19	protein_coding	1.8e-02	-1.04	4.8e-02	-0.96	0.08	–
74ENST00000373109	SPOCK2	37	protein_coding	1.5e-02	1.04	4.3e-02	0.94	-0.10	–
74ENST00000590935	DUSP3	21	protein_coding	6.1e-03	-1.04	5.1e-02	-0.70	0.34	–
74ENST00000409817	CXCR4	27	protein_coding	2.0e-02	1.04	5.1e-02	0.97	-0.07	YY
74ENST00000581082	PER1	30	protein_coding	2.0e-02	1.04	1.4e-01	0.66	-0.38	YY
74ENST00000446643	WDR43	13	protein_coding	4.6e-02	-1.04	1.6e-01	-0.82	0.22	–
74ENST00000378474	MID1IP1	20	protein_coding	3.3e-02	-1.03	1.2e-01	-0.85	0.19	–
74ENST00000585770	MRM1	20	protein_coding	1.8e-02	-1.03	6.0e-02	-0.87	0.16	Y-
74ENST00000427972	UTP14A	9	protein_coding	1.1e-02	-1.03	4.3e-02	-0.86	0.17	–
74ENST00000399107	POLR3G	6	protein_coding	1.4e-02	-1.03	5.5e-02	-0.83	0.20	–
74ENST00000517668	ST3GAL1	9	protein_coding	1.4e-02	-1.03	5.1e-02	-0.83	0.19	–
74ENST00000360128	MAK16	33	protein_coding	2.0e-02	-1.02	1.2e-01	-0.71	0.31	–
74ENST00000438527	DDX31	24	protein_coding	1.9e-02	-1.02	5.8e-02	-0.89	0.13	–
74ENST00000359013	TGFBR2	23	protein_coding	2.2e-02	1.02	1.1e-01	0.77	-0.25	Y-
74ENST00000441717	JAM3	24	protein_coding	2.3e-02	1.01	1.3e-01	0.71	-0.31	Y-
74ENST00000506073	TXK	10	protein_coding	1.7e-02	1.01	1.3e-01	0.63	-0.38	YY
74ENST00000361507	GAB2	30	protein_coding	3.9e-02	1.01	1.7e-01	0.74	-0.27	Y-
74ENST00000384596	Y_RNA	25	misc_RNA	2.7e-02	-1.01	6.6e-02	-0.95	0.05	–
74ENST00000404843	ASXL2	22	protein_coding	1.8e-02	1.00	6.2e-02	0.84	-0.16	–
74ENST00000555638	TMEM229B	9	protein_coding	4.9e-02	-1.00	1.6e-01	-0.80	0.20	–

Supplementary Table S2: Significantly differentially expressed genes between 6 hours DEX and EtHO treated cells. Values for DEX and PRED treatments are shown in columns with suffix D and P, respectively. Column $M.PvsD$ displays the difference in GC-regulation between PRED and DEX treatment. Column GBR contains ChIP-chip determined GR-DNA interaction information from [5] (with *YY* and *Y-* indicating an identified GR-DNA interaction in their promoter region of the gene with or without the presence of a GR-response element, respectively; – represents genes without a GR-DNA interaction and an empty cell is shown for genes that could not be detected by ChIP-chip. Genes not highlighted have been found significantly regulated by DEX as well as PRED treatment. Blue coloring highlights genes which don't meet the regulation ($|M| > 1$), but satisfy the significance cut-off ($p_{BH} < 0.05$) in PRED treated samples. Green indicates genes which don't meet the significance cut-off ($p_{BH} < 0.05$) while passing the regulation cut-off in PRED treatment. Red coloring indicates genes which are not significantly regulated upon PRED exposure(i.e. have an $|M| < 1$ and a $p_{BH} > 0.05$).

Probe set ID	Gene name	No. probes	Gene biotype	$p_{BH}.D$	$M.D$	$p_{BH}.P$	$M.P$	$M.PvsD$	GBR
74ENST00000540964	TLR5	24	protein_coding	6.1e-03	1.56	1.5e-01	0.69	-0.86	–
74ENST00000489474	RPS18	4	protein_coding	3.6e-02	-1.31	3.6e-01	-0.57	0.74	
74ENST00000581301	MRM1	7	protein_coding	3.2e-02	-1.23	2.5e-01	-0.65	0.58	
74ENST00000428982	SYNGAP1	22	protein_coding	1.6e-02	1.17	1.8e-01	0.61	-0.56	–
74ENST00000393409	PLXNA1	36	protein_coding	1.3e-02	1.15	3.0e-01	0.41	-0.75	–
74ENST00000515965	RNU6-377P	24	snRNA	1.9e-02	-1.13	1.9e-01	-0.59	0.55	–
74ENST00000391143	SNORA48	25	snoRNA	2.3e-02	-1.12	1.7e-01	-0.69	0.43	–
74ENST00000416587	RNF144A	9	protein_coding	2.6e-02	1.11	2.0e-01	0.62	-0.49	–
74ENST00000461853	RN7SL173P	25	misc_RNA	9.1e-03	-1.11	5.1e-01	-0.23	0.88	–
74ENST00000381293	IL6ST	13	protein_coding	7.7e-03	1.09	1.7e-01	0.48	-0.61	–
74ENST00000543175	PTRH1	18	protein_coding	2.1e-02	1.09	2.0e-01	0.58	-0.51	–
74ENST00000371222	JUN	32	protein_coding	9.7e-03	1.09	1.1e-01	0.65	-0.44	–
74ENST00000330777	SGK223	30	protein_coding	1.6e-02	1.08	1.2e-01	0.67	-0.41	–
74ENST00000544928	AL021707.2	3	pseudogene	2.9e-02	-1.07	3.7e-01	-0.43	0.65	
74ENST00000395071	SYNGAP1	27	protein_coding	2.0e-02	1.07	1.5e-01	0.66	-0.41	
74ENST00000365352	Y_RNA	24	misc_RNA	2.1e-02	-1.06	4.2e-01	-0.34	0.72	–
74ENST00000374557	EPB41L4B	21	protein_coding	1.4e-02	1.06	1.5e-01	0.58	-0.48	–
74ENST00000404926	RP3-415N12.1	14	pseudogene	4.1e-02	-1.05	5.0e-01	-0.35	0.71	–
74ENST00000602634	TSNAX-DISC1	49	protein_coding	1.6e-02	1.05	1.2e-01	0.69	-0.36	
74ENST00000581082	PER1	30	protein_coding	2.0e-02	1.04	1.4e-01	0.66	-0.38	YY
74ENST00000506073	TXK	10	protein_coding	1.7e-02	1.01	1.3e-01	0.63	-0.38	YY

Supplementary Table S3: Significantly differentially expressed genes between DEX and EtOH treated cells, which are regulated with an absolute M-value smaller than 0.7 by PRED treatment.

Probe set ID	Gene name	No. probe	Gene biotype	$p_{BH}.D$	$M.D$	$p_{BH}.P.low$	$M.P.low$	$p_{BH}.P.high$	$M.P.high$
74ENST00000342210	TLR5	21	protein_coding	4.0e-02	0.59	4.4e-01	0.29	2.2e-01	0.27
74ENST00000489474	RPS18	4	protein_coding	6.7e-01	-0.29	2.5e-01	-0.87	9.9e-01	-0.00
74ENST00000581301	MRM1	7	protein_coding	9.3e-01	-0.04	3.8e-01	-0.33	9.8e-02	-0.39
74ENST00000428982	SYNGAP1	22	protein_coding	4.9e-01	0.27	5.5e-01	0.31	2.8e-01	0.30
74ENST00000393409	PLXNA1	36	protein_coding	7.7e-03	1.10	5.3e-01	0.34	1.5e-02	0.99
74ENST00000515965	RNU6-377P	24	snRNA	4.2e-01	-0.24	8.0e-01	-0.12	7.9e-01	0.06

Probe set ID	Gene name	No. probe	Gene biotype	$p_{BH}.D$	$M.D$	$p_{BH}.P.low$	$M.P.low$	$p_{BH}.P.high$	$M.P.high$
74ENST00000391143	SNORA48	25	snoRNA	4.3e-01	-0.29	9.1e-01	-0.07	8.4e-02	-0.49
74ENST00000416587	RNF144A	9	protein_coding	4.9e-02	0.73	1.6e-01	0.65	1.1e-01	0.49
74ENST00000461853	RN7SL173P	25	misc_RNA	5.4e-01	-0.17	4.7e-01	-0.25	3.0e-01	0.20
74ENST00000381293	IL6ST	13	protein_coding	1.1e-02	1.13	1.2e-01	0.82	2.0e-01	0.42
74ENST00000543175	PTRH1	18	protein_coding	3.7e-02	0.67	5.2e-01	0.28	5.0e-02	0.56
74ENST00000371222	JUN	32	protein_coding	4.2e-04	1.42	7.5e-02	0.70	2.2e-02	0.72
74ENST00000330777	SGK223	30	protein_coding	5.2e-02	0.65	3.9e-01	0.37	4.6e-02	0.60
74ENST00000544928	AL021707.2	3	pseudogene	6.1e-01	-0.30	1.4e-01	-0.97	7.1e-01	0.16
74ENST00000395071	SYNGAP1	27	protein_coding	5.3e-01	0.24	5.9e-01	0.27	3.5e-01	0.25
74ENST00000365352	Y_RNA	24	misc_RNA	3.7e-01	0.28	3.3e-01	0.38	6.8e-01	0.10
74ENST00000374557	EPB41L4B	21	protein_coding	9.7e-01	-0.02	6.4e-01	-0.21	7.6e-01	0.08
74ENST00000404926	RP3-415N12.1	14	pseudogene	9.8e-01	-0.02	8.9e-01	-0.15	4.9e-01	-0.31
74ENST00000602885	TSNAX-DISC1	45	protein_coding	4.2e-03	1.24	2.2e-01	0.63	7.7e-02	0.59
74ENST00000317276	PER1	32	protein_coding	3.7e-02	0.80	3.3e-01	0.49	1.5e-02	0.96
74ENST00000506073	TXK	10	protein_coding	9.3e-01	0.07	9.8e-01	-0.03	4.6e-01	-0.30

Supplementary Table S4: GC-regulation of significantly differentially expressed genes between DEX and EtOH treated cells regulated with an absolute M-value smaller than 0.7 by PRED treatment in a second microarray data set with DEX treated C7H2 cells and C7H2 cells treated with two different concentrations of the water soluble prednisolone Solu-dacortin [prednisolone 21-(sodium succinate)]. Columns with suffix D contain values for DEX treated samples, $P.low$ and $P.high$ values for 4.0 and 8.0e-6M Solu-dacortin.

Ensembl Gene ID	Gene name	No. probes	$p_{BH}.D$	$M.D$	$p_{BH}.P$	$M.P$	GBR
ENSG00000157514	TSC22D3	14	2.1e-05	6.21	2.6e-05	6.06	YY
ENSG00000185338	SOCS1	16	1.4e-04	3.52	6.3e-04	2.89	YY
ENSG00000159200	RCAN1	14	1.0e-03	2.45	7.5e-03	1.85	YY
ENSG00000096060	FKBP5	17	5.6e-04	2.11	7.8e-04	2.11	YY
ENSG00000113580	NR3C1	17	9.8e-04	1.54	1.2e-03	1.55	–
ENSG00000168209	DDIT4	30	6.0e-03	1.32	1.3e-02	1.25	–

Supplementary Table S5: GC-regulation of some known GR target genes. Values for DEX and PRED treatments are shown in columns with suffix D and P, respectively. Column GBR contains ChIP-chip determined GR-DNA interaction information from [5] (with *YY* and *Y-* indicating an identified GR-DNA interaction in their promoter region of the gene with or without the presence of a GR-response element, respectively; – represents genes without a GR-DNA interaction and an empty cell is shown for genes that could not be detected by ChIP-chip.

Bibliography

(1) B. Oppl, A. Kofler, S. Schwarz, J. Rainer and R. Kofler, *Wiener klinische Wochenschrift*, Apr. 2011, **123**, 222–229.

(2) J. L. McNeer and J. B. Nachman, *British journal of haematology*, June 2010, **149**, 638–652.

(3) G. J. L. Kaspers, G. J. Kaspers, A. J. P. Veerman, A. J. Veerman, C Popp-Snijders, M Lomecky, C. H. Van Zantwijk, L. M. J. W. Swinkels, L. M. Swinkels, E. R. Van Wering and R Pieters, *Medical and Pediatric Oncology*, Aug. 1996, **27**, 114–121.

(4) J Styczyński, M Wysocki, W Balwierz, R Rokicka-Milewska, M Matysiak, A Balcerska, A Kowalczyk, J Wachowiak, D Sońta-Jakimczyk and A Chybicka, *Neoplasma*, 2002, **49**, 178–183.

(5) J. Rainer, J. Lelong, D. Bindreither, C. Mantinger, C. Ploner, S. Geley and R. Kofler, *Molecular endocrinology (Baltimore, Md)*, Jan. 2012, **26**, 178–193.
